# Genome-Wide Identification and Characterization of SPX Domain-Containing Members and Their Responses to Phosphate Deficiency in *Brassica napus*

**DOI:** 10.3389/fpls.2017.00035

**Published:** 2017-01-25

**Authors:** Hongyuan Du, Chang Yang, Guangda Ding, Lei Shi, Fangsen Xu

**Affiliations:** ^1^National Key Laboratory of Crop Genetic Improvement, Huazhong Agricultural UniversityWuhan, China; ^2^Key Laboratory of Arable Land Conservation, Ministry of Agriculture, Huazhong Agricultural UniversityWuhan, China

**Keywords:** *Brassica napus*, genome-wide analysis, SPX domain gene, Pi stress, expression profile, *cis*-element, gene function

## Abstract

The importance of SPX domain-encoding proteins to phosphate (Pi) homeostasis and signaling pathways has been well-documented in rice and *Arabidopsis*. However, global information and responses of SPX members to P stress in allotetraploid *Brassica napus*, one of the world’s major oil crops that is sensitive to P deficiency, remain undefined. We identified a total of 69 SPX domain-containing genes in the *B. napus* genome. Based on the domain organizations, these genes were classified into four distinct subfamilies—SPX (11), SPX-EXS (43), SPX-MFS (8), and SPX-RING (7)—that represented clear orthologous relationships to their family members in *Arabidopsis*. A *cis*-element analysis indicated that 2 ∼ 4 P1BS elements were enriched in the promoter of SPX subfamily genes except *BnaSPX4s*. RNA-Seq analysis showed that *BnaSPX* genes were differentially expressed in response to Pi deficiency. Furthermore, quantitative real-time reverse transcription PCR revealed that nine SPX subfamily genes were significantly induced by Pi starvation and recovered rapidly after Pi refeeding. A functional analysis of two paralogous *BnaSPX1* genes in transgenic *Arabidopsis* indicated their functional divergence during long-term evolution. This comprehensive study on the abundance, molecular characterization and responses to Pi deficiency of *BnaSPX* genes provides insights into the structural and functional diversities of these family members in *B. napus* and provides a solid foundation for future functional studies of *BnaSPX* genes.

**Highlight:** The genome-wide identification and characterization of SPX genes in *B. napus* and their responses to Pi deficiency provide comprehensive insights into the structural and functional diversities of the family members in *B. napus* and their potential in Pi homeostasis and signaling responsiveness to Pi stress.

## Introduction

Phosphorus (P), an indispensable macronutrient for plant growth and development, is absorbed by root systems in the form of Pi from soil matrices (Nussaume et al., 2011). However, Pi in soils is dilute and less diffusible because it mainly exists as an integral component in Ca, Fe or Al salts, or in the form of organic molecules (Raghothama and Karthikeyan, 2005). Therefore, plants often encounter a scarcity of Pi in soils in both agricultural and natural contexts. However, plants have gained series of evolutionarily adapted strategies to actively interact with Pi stress (Vance et al., 2003; Morcuende et al., 2007; Jain et al., 2012). Unveiling the molecular mechanism underlying the Pi starvation responses of plants would be pivotal for developing crop plants with enhanced Pi acquisition and better P use efficiency under low Pi conditions. Considerable information has been gathered on the components of the Pi starvation signaling pathway that has been well reviewed in the past decade (Chiou and Lin, 2011; Wu et al., 2013; Zhang et al., 2014; Baker et al., 2015); increasing studies have established the importance of SPX domain-containing proteins in Pi homeostasis and Pi signaling. To date, the identification and characterization of SPX members has been completed in many plant species including *Arabidopsis* (Duan et al., 2008), soybeans (Yao et al., 2014a), common beans (Yao et al., 2014b), and rice (Wang et al., 2009, 2014; Liu et al., 2010; Shi et al., 2014).

The SPX domain (Pfam PF03105) was named after the Suppressor of Yeast gpa1 (Syg1), the yeast Phosphatase 81 (Pho81), and the human Xenotropic and Polytropic Retrovirus receptor 1 (Xpr1). This hydrophilic domain of the SPX proteins is found at the N-termini of various proteins. In plants, proteins harboring the SPX domain are classified into four groups based on the presence of additional structural domains, namely, the SPX, SPX-EXS, SPX-MFS, and SPX-RING subfamilies (Secco et al., 2012). There are 20 and 14 SPX members in *Arabidopsis* and rice, respectively, which are different in structure, expression, protein subcellular location, and gene function (Duan et al., 2008; Secco et al., 2012).

Proteins exclusively harboring the SPX domain are referred to as SPX subfamily members. In *Arabidopsis* and rice, the SPX subfamily consists of four and six members, named AtSPX1-AtSPX4 and OsSPX1-SPX6, respectively (Duan et al., 2008; Secco et al., 2012). All SPX genes, with the exception of AtSPX4 and OsSPX4, are PSI. The major roles of SPX subfamily genes are to modulate the activities of PHR1 in the regulation of PSI gene transcription, either by controlling the translocation of PHR1 from the cytoplasm to the nucleus (Lv et al., 2014) or by competing with the P1BS element for binding to PHR1 (Puga et al., 2014; Wang et al., 2014).

The second subfamily of SPX domain-containing proteins is associated with the EXS domain (PF03124), which is embedded in a hydrophobic region and named after the yeast ERD1, the human XPR1 and the yeast SYG1 proteins (Hamburger et al., 2002). The PHO1 family members are the only proteins in eukaryotes that contain both the SPX and EXS domains (Wang et al., 2004). Transient expression analysis in *Nicotiana benthamiana* indicated that the EXS domain of PHO1 is essential for Pi export activity and proper localization to the Golgi and *trans*-Golgi network, although the EXS domain by itself cannot mediate Pi export (Wege et al., 2016). The genome of *Arabidopsis* contains 11 members of the PHO1 gene family (designated as PHO1 and PHO1; H1 to PHO1; H10). Diverse tissue expression patterns implied a broad role for the PHO1 gene family. However, to date, only AtPHO1 and AtPHO1;H1 have been studied and found to be involved in Pi homeostasis (Stefanovic et al., 2007, 2011). Bioinformatics and phylogenetic analysis showed that the rice genome has three PHO1 homologs that cluster with AtPHO1 and AtPHO1;H1. In contrast to the *Arabidopsis* PHO1 gene family, all three rice PHO1 genes have a *cis*-natural antisense transcript located at the 5′ end of the genes. OsPHO1;2 played a key role in the transfer of Pi from roots to shoots in rice and could be regulated by its *cis*-natural antisense transcripts (Secco et al., 2010).

The third subfamily of SPX domain-possessing proteins is the SPX-MFS (Major Facility Superfamily) family (PF07690). There are three SPX-MFS members in the genomes of both *Arabidopsis* and rice, namely SPX-MFS1 to SPX-MFS3, which are also designated the PHOSPHATE TRANSPORTER 5 (PHT5) family in *Arabidopsis* (Secco et al., 2012; Wang et al., 2012; Liu et al., 2016). Overexpression of PHT5 leads to Pi over accumulation and retarded growth. Based on ^31^P-magnetic resonance spectroscopy analysis, *Arabidopsis* pht5;1 loss-of-function mutants accumulate less Pi and exhibit a lower vacuolar-to-cytoplasmic Pi ratio than controls. OsSPX-MFS1 and OsSPX-MFS3 were suppressed by Pi deficiency, whereas OsSPX-MFS2 was induced (Lin et al., 2010). Heterologous complementation of a yeast mutant impaired in Pi transport indicated the capacity of OsSPX-MFS1 to transport Pi. Mutated OsSPX-MFS1 alters Pi re-mobilization in rice (Wang et al., 2012). OsSPX-MFS3 is a low affinity Pi transporter that mediates Pi eﬄux from the vacuole into the cytosol and is coupled to proton movement (Wang et al., 2015).

The fourth SPX protein subfamily contains an additional domain named the Really Interesting New Gene (RING) finger domain, a specialized type of zinc finger domain that is involved in the mediation of protein–protein interactions. The presence of a RING finger domain is a characteristic of RING-class E3 ubiquitin protein ligases, which are capable of transferring ubiquitin from an E2 enzyme to a substrate protein. Only two proteins in both rice and *Arabidopsis* possess the RING and SPX domains (Secco et al., 2012). To date, the only characterized member of the SPX-RING family is also called Nitrogen Limitation Adaptation (NLA), first identified by its role in nitrogen starvation resistance (Peng et al., 2007). Later, a combination of physiology and genetic studies demonstrated that NLA regulates nitrate-dependent Pi homeostasis in *Arabidopsis* (Kant et al., 2011). Phosphate analysis revealed that the *atnla* mutant showed increased Pi uptake and Pi content, especially under low-nitrate and high-phosphate availability, relative to WT plants.

Allotetraploid rapeseed (*Brassica napus*, A_n_A_n_C_n_C_n_, 2*n* = 38, 840 Mb) originated from a natural hybridization between *B. rapa* (A_r_A_r_, 2*n* = 20, 312 Mb) and *B. oleracea* (C_o_C_o_, 2*n* = 18, 540 Mb) approximately 7,500–12,500 years ago (Nagaharu, 1935; Chalhoub et al., 2014). Through long-term evolution and domestication, rapeseed has become second-leading crop source of vegetable oil (following soybean) for human consumption, with a total production of over 60 million tons worldwide (Bancroft et al., 2011). Although SPX domain-containing proteins have been characterized in well-studied model plants, such as rice and *Arabidopsis*, systematic descriptions of evolution, distribution and origin of the SPX gene family have not been conducted in *B. napus*, which is sensitive to P deficiency. In this study, we first identified all putative SPX genes in the *B. napus* genome, systematically characterized their structural features and conserved domains, and then provided RNA-Seq-based gene expression profiles and qRT-PCR data for *BnaSPX* genes both in the roots and shoots of *B. napus* in response to Pi stress. Our analyses provide the framework for future functional studies of this important gene family and the basis for exploiting candidate genes for genetic engineering of P efficiency in *B. napus.*

## Materials and Methods

### Identification of SPX Genes in *B. napus*

SPX members were identified in *B. napus* based on homology with the 20 known SPX protein sequences from *Arabidopsis* (Secco et al., 2012) using the BLAST search program in the CNS – Genoscope database^1^ (Chalhoub et al., 2014). After removing redundant sequences and incomplete ORF sequences, SMART tools^2^ (Letunic et al., 2014), NCBI Conserved Domain Search database^3^ (Marchler-Bauer et al., 2015), and InterProScan^4^ (Mitchell et al., 2015) were used to confirm the presence of characterized domains in the candidate sequences. The putative members of the SPX family and their gene sequences were identified and defined for further analyses. The number of amino acids, CDS lengths and chromosome locations of the *BnaSPX* genes were obtained from the *B. napus* database. Physicochemical parameters, including the molecular weight (kDa) and pI of each *BnaSPX* protein, were calculated using the compute pI/Mw tool in ExPASy^5^ (Gasteiger et al., 2003). GRAVY values were calculated using the PROTPARAM tool^6^ (Gasteiger et al., 2003). Subcellular location prediction was conducted using Wolf Psort^7^ (Hamburger et al., 2007), and the TargetP1.1^8^ (Emanuelsson et al., 2007) server. The synteny relationships between the *BnaSPXs* and SPX genes in *A. thaliana, B. rapa*, and *B. oleracea* were evaluated using the search syntenic genes tool in BRAD^9^ (Cheng et al., 2012).

### Multiple Alignment and Phylogenetic Analysis of *BnaSPX* Family Genes

Multiple sequence alignment of all predicted BnaSPX protein sequences (69) and AtSPXs protein sequences (20) was performed using Clustal W software, and an un-rooted phylogenetic tree of the 89 full-length SPX protein sequences was generated with neighbor-joining (NJ) criteria in MEGA 6 (Tamura et al., 2013) with 1000 bootstrap replicates.

### Gene Structure and Conserved Motif Analysis of BnaSPX Members

Multiple Expectation Maximization for Motif Elicitation^10^ (Bailey et al., 2009) was used to identify the conserved motif structures encoded by the *BnaSPX* family genes. Parameter setting included output motifs (15), minimum motif width (12), and maximum motif width (165). The exon-intron structures of the *BnaSPX* family genes were investigated using the online Gene Structure Display Server^11^ (Hu et al., 2015) based on full-length CDS alignments with corresponding genomic sequences.

### Plant Materials and Treatments

The rapeseed genotype Eyou Changjia was used for gene cloning and expression analysis. The plants were grown hydroponically in an illuminated culture room with a cycle of 16 h/24°C day and 8 h/22°C night, and a light intensity of 300–320 μmol proton m^-2^ s^-1^. Six-day-old seedlings germinated on gauze moistened with deionized water were transferred to nutrient solution. In addition to the P, the complete basal nutrient solution contained Ca(NO_3_)_2_ 4.5 mM, KNO_3_ 4.0 mM, MgSO_4_ 2.0 mM, H_3_BO_3_ 46.0 μM, MnCl_2_ 9.0 μM, CuCl_2_ 0.3 μM, ZnCl_2_ 0.8 μM, Na_2_MoO_4_ 0.1 μM, and EDTA-Fe 50.0 μM (Yang et al., 2011). The initial nutrient solution was 1/4 full-strength for the first 5 days, then nutrient solution was replaced with half-strength and full-strength solution at intervals of 5 days. To study gene expression patterns during Pi starvation and resupply, the seedlings were first grown under 250 μM Pi (KH_2_PO_4_) for 15 days after transplanting, then the seedlings were transferred to Pi-free solution, and the leaves and roots were sampled after 0, 12, 72, and 240 h. The plants grown in the Pi-free solution for 10 days (240 h) were retransferred to Pi-sufficient solution (250 μM Pi), and then the leaves and roots were harvested at 6 and 72 h after transferred. To analyze the expression patterns of *BnaSPX* members in different tissues under Pi-deficiency condition, a pot culture was performed with 10 mg P per kg soil. During full-bloom stage, old leaf (the lower third leaf), young leaf (the upper first leaf), bud, flowers, pods, and pod peduncle were harvested for RNA extraction. Three biological replicates were included in all experiments and all samples were frozen in liquid nitrogen and stored at -80°C prior to RNA extraction.

For phenotype tests of transgenic lines, the *Arabidopsis* seeds were surface sterilized and cold treated at 4°C for 2 days. Then, the seeds were germinated on MGRL medium containing 1% (w/v) sucrose and 0.9% (w/v) agar with high Pi (1 mM Pi, HP) and low Pi (50 μM Pi, LP) treatments.

### Expression Pattern Analysis of *BnaSPXs* in Response to Pi Starvation using RNA-Seq Data

For the RNA-Seq experiment, rapeseed seedlings were grown hydroponically under 250 μM Pi (high P, HP) for 15 days, then half the seedlings were moved to P-free nutrient solution (-P, 0 μM P) and the remaining seedlings were maintained under HP conditions and used as control. The roots and leaves of every individual seedling were harvested at 10 days after the P was removed (KH_2_PO_4_ was replaced by K_2_SO_4_). Three biological replicates were included. Total RNA was extracted from plant samples using TRIzol reagent according to the manufacturer’s instructions (Invitrogen, USA). The quality and integrity of the total RNA were assessed using a NanoDrop 2000 spectrophotometer (Thermo Scientific, USA). Three microgram of RNA per sample was further processed by the purification of polyA-containing mRNA, mRNA fragmentation, double-stranded cDNA synthesis, and polymerase chain reaction (PCR) amplification using the Illumina TruSeq RNA Sample Preparation Kit (Illumina, USA) according to the manufacturer’s protocol. The final cDNA libraries were sequenced on an Illumina HiSeqTM 2000 platform. For the RNA-Seq analysis based on the raw reads, clean reads were first generated by removing adapter sequences, low-quality reads and uncertain bases. A total of 643,846,484 raw reads and 571,929,682 clean reads were generated. The mapped reads were 496,073,698 and the unique mapped reads were 430,982,901. So the unique mapped rate (%) was 75.40%, which were mapped to the reference genome (version 5^12^) by TopHat2 using default settings. To measure the level of gene expression, the FPKM value of each gene was calculated based on the length of the gene and the read count mapped to this gene. False discovery rate (FDR) was used in multiple hypotheses to correct the results for *P*-value. In the present study, FDR ≤ 0.05 and the absolute value of log2 (fold-change) ≥1 were used as the threshold to screen the differentially expressed genes (DEGs). A heat map of *BnaSPXs* gene expression was generated based on the FPKM values using the Multiexperiment View software. FPKM values based on transcriptome data are shown in Supplementary Table S3.

### Expression Analysis by qRT-PCR

Total RNA was extracted from plant samples using TRIzol reagent according to the manufacturer’s instructions (Invitrogen, USA). First-strand cDNA was synthesized using M-MLV reverse transcriptase and oligo (dT) according to the manufacturer’s protocol (Promega, USA). Quantitative real-time RT-PCR was performed on a CFX96 Real-Time PCR Detection System (Bio-Rad, USA) with the SYBR Green system (Toyobo, Japan). The *BnaActin2* gene was used as an internal control, and the fold change was analyzed via the 2^-ΔΔCT^ method (Livak and Schmittgen, 2001). The gene-specific primers used in the qRT-PCR analysis were listed in Supplementary Table S4.

### Characterization of Putative *Cis*-elements in the Promoter Region of the SPX Genes in *B. napus*

The 1500 bp upstream sequences relative to the translation start codon of SPX genes were downloaded from CNS – Genoscope^12^. The upstream 1500 bp regions of *BnaSPX* genes were analyzed to determine the *cis*-regulatory elements using the plant *cis*-element database PlantCARE^13^ Lescot et al., 2002).

### Vector Construction and Plant Transformation

To construct the p*BnaA2.SPX1*:BnaA2.SPX1 and p*BnaC3.SPX1*:*BnaC3.SPX1* vectors, the genomic sequences of *BnaA2.SPX1* and *BnaC3.SPX1*, together with their upstream 1500 bp sequences, were amplified from the genomic DNA (gDNA) isolated from rapeseed genotype Eyou Changjia seedlings. The sequence-confirmed PCR fragments were inserted into the binary vector pBin35SRed using *Asc*I and *Xba*I restriction enzymes. The resulting vectors carried a DsRed fluorescent protein as a selectable marker for transgenic lines (Pidkowich et al., 2007). Semi-RT-PCR was performed to validate the expression level of exogenous SPX1 in *Arabidopsis* wild-type (WT) Col-0 and transgenic plants by using specific primers. The primer information was present in the Supplementary Table S4.

For subcellular localization analysis, the CDSs (without the stop codon) of *BnaA3.SPX1, BnaA2.SPX1, BnaC3.SPX1, BnaA3.SPX2*, and *BnaC3.SPX2* were PCR amplified from rapeseed cDNA and fused to the 5′ terminus of GFP in the vector pMDC83 via *Pac*I and *Asc*I restriction enzyme sites to generate 35S-*BnaSPXs*-GFP vectors.

The primers used for vector construction are listed in Supplementary Table S4. All constructs were mobilized into the *Agrobacterium tumefaciens* strain GV3101. Recombinant vectors p*BnaA2.SPX1*:BnaA2.SPX1 and p*BnaC3.SPX1*:*BnaC3.SPX1* were transformed into *Arabidopsis* via the *Agrobacterium*-mediated flower dip method (Clough and Bent, 1998), and 35S-*BnaSPXs*-GFP were used for transient expression in the leaves of tobacco (*N. benthamiana*).

### Sub-Cellular Localization Analysis

The *Agrobacterium* strains harboring 35S-*BnaA2.SPX1-GFP*, 35S-*BnaA3.SPX1-GFP* 35S-*BnaC3.SPX1-GFP*, 35S-*BnaA3.SPX2-GFP*, and 35S-*BnaC3.SPX2-GFP*, respectively, were grown to a cell density of OD_600_ = 0.8 and then harvested and resuspended in an infiltration buffer (0.5 M MES (pH5.6), 100 mM AS and 10 mM MgCl_2_) to the same concentration. Then, the suspension liquid and DAPI solution (5 mg/mL) were infiltrated into the lower epidermal side of 25-day-old leaves of *N. benthamiana* plants. Two days after the injection, the GFP fluorescence and DAPI staining were observed using a confocal laser scanning microscope (TCS SP2, Leica). All fluorescence experiments were independently repeated at least three times.

### Quantification of Total P

The wild type and transgenic lines were raised in petri plates containing HP medium (1 mM) or LP (50 μM) for 11 days, then the shoots and roots were harvested for measurement of total P content as described (Ramaiah et al., 2014). Three biological replicates were included in the experiment. The values are expressed as total P mmol^-1^ tissue dry weight.

### Statistical Analysis

Data were analyzed using two-way analysis of variance (ANOVA) to detect statistically significant differences in the results of dry weight, total P concentration and qRT-PCR analysis. The data generated were subjected to Tukey’s test, and different letters on the histograms indicated mean values that were statistically different at *P* ≤ 0.05.

## Results

### Genome-Wide Identification of SPX Members in *B. napus*

Genes encoding SPX domain-containing proteins were identified in the *B. napus* genome by employing homology searches and domain confirmation analysis, and a total of 69 SPX genes (*BnaSPXs*) were identified in the genome of *B. napus*. Based on the presence of additional domains in protein structure, the 69 *BnaSPX* genes were classified into four subfamilies, including SPX, SPX-EXS, SPX-MFS, and SPX-RING. Among these four subfamilies, the SPX-EXS group is the largest with 43 members, and the SPX, SPX-MFS, and SPX-RING subfamilies include 11, 8, and 7 members, respectively. The details of all 69 rapeseed SPX members, including the number of exons, protein properties, GRAVY values and predicted subcellular locations, are listed in Supplementary Table S1. The rapeseed SPX proteins consist of 239–825 amino acids, with corresponding molecular weights ranging from 27.61 to 95.14 kDa. BnaSPXs of the SPX-EXS and SPX-MFS subfamilies contain more amino acid residues than those of SPX and SPX-RING. EXPASY analysis revealed that the SPX protein sequences largely varied in isoelectric point (pI) values ranging from 4.91 to 9.54, but most proteins in the same subfamily have similar parameters. Almost all SPX-EXS and SPX-RING proteins have relatively high isoelectric points (pI > 7), whereas the remaining proteins, particularly those in the SPX-MFS family, have pI < 7. GRAVY value was defined by the sum of the hydropathy values of all amino acids divided by the protein length. Except for the proteins in the SPX-MFS subfamily, nearly all of the BnaSPXs are hydrophilic, with a GRAVY value <0. TargetP and Wolf Psort were used to predict the subcellular location of the 69 BnaSPX proteins, which included the nucleus, plasma membrane and cytoplasm. Most of the proteins in the SPX-MFS and SPX-EXS subfamilies are located in the plasma membrane, and nearly all the members in the SPX and SPX-RING groups are exclusively located in the nucleus. The diversity in subcellular locations implies different functions within the SPX family. The 67 SPX genes (except for *BnaCn.SPX-MFS3* and *BnaCn.PHO1; H8*) are unevenly distributed on 18 of *B. napus* 19 chromosomes. The majority of *BnaSPX* genes are located on the chromosome arms that are associated with high rates of recombination. The number of SPX genes on a single *B. napus* chromosome ranges from 1 (A04) to 11 (A09) (**Supplementary Figure S1**). To get a better understanding of the *BnaSPXs* gene evolution mechanism, we searched for the syntenic genes of *BnaSPXs* gene with other *Brassicaceae* species. The synteny analysis demonstrated that most SPX gene family members are located in well-conserved synteny regions, and some genes were deleted or gained. These findings indicate that some genes might have been translocated into a non-syntenic region (Supplementary Table S2).

### Sequence Alignment and Phylogenetic Analysis of *BnaSPX* Genes

**FIGURE 1:**
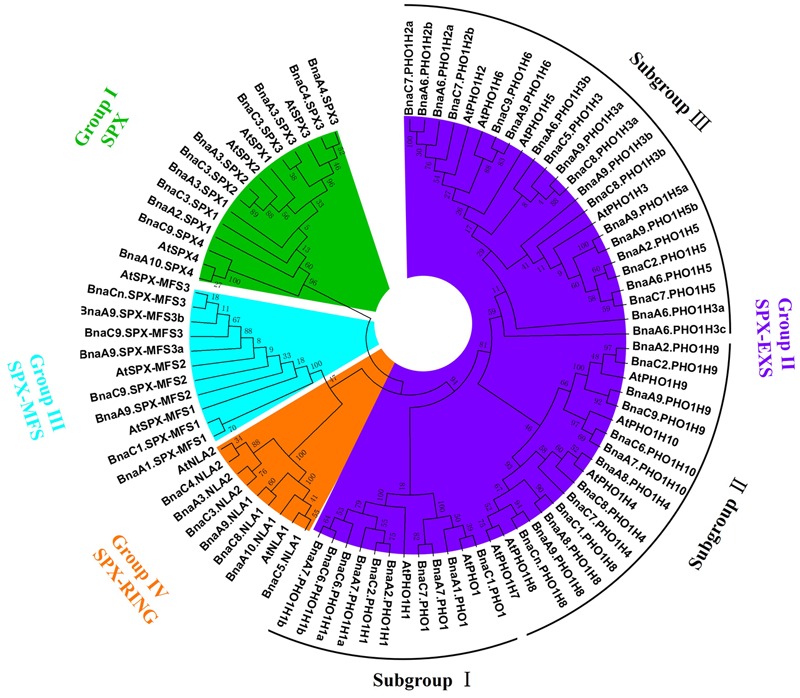
**Phylogenetic tree of all SPX proteins from *Brassica napus* and *Arabidopsis*.** The unrooted tree was generated by MEGA 6 with the full-length amino acid sequences of the 69 *B. napus* SPX proteins and 20 *Arabidopsis* SPX proteins using the neighbor-joining (NJ) method with 1000 bootstrap replicates. Four SPX subfamilies (groups), including SPX, SPX-EXS, SPX-MFS, and SPX-RING, were distinguished by different colors.

To examine the phylogenetic relationships among the SPX domain-containing proteins in *B. napus*, an unrooted phylogenetic tree was constructed from alignments of the full-length SPX amino acid sequences using the NJ method (**Figure 1**). The phylogenetic tree confirms that *BnaSPXs* could be classified into four groups (subfamilies), which is consistent with the classification results obtained using homology searches as described above (Supplementary Table S1). The largest group SPX-EXS was further classified into three distinct clades (**Figure 1**). Clade I genes are homologous to the *Arabidopsis PHO1* and *PHO1;H1*, the only two genes known to be involved in long-distance Pi transport (Stefanovic et al., 2007). Clade II genes are homologous to *PHO1;H4, PHO1;H7, PHO1;H8, PHO1;H9*, and *PHO1;H10*. Clade III genes are homologous to *PHO1;H2, PHO1;H3, PHO1;H5*, and *PHO1;H6* in *Arabidopsis*.

### Structural and Conserved Motif Analysis of *BnaSPX* Genes

**FIGURE 2:**
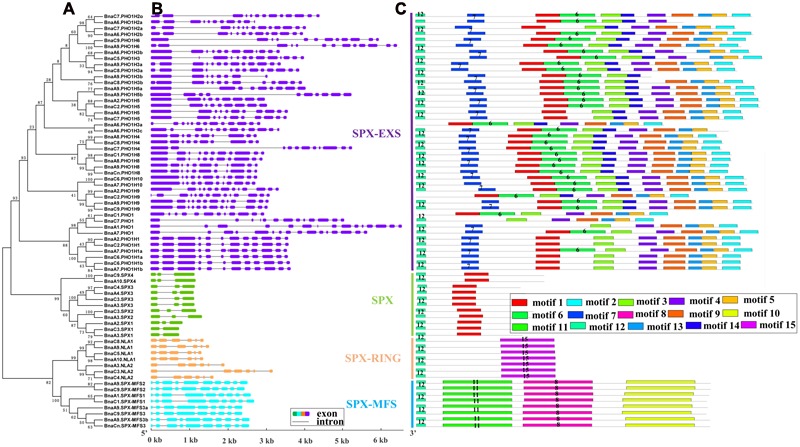
**Schematic representations of the exon–intron compositions and conserved motifs of the *BnaSPX* genes. (A)** NJ phylogenetic tree of *BnaSPXs*. **(B)** Exon-intron organization of *BnaSPX* genes. Double-sided wedge boxes represent exons and gray lines represent introns. Different colors indicate different *SPX* gene subfamilies. The exon and intron sizes can be estimated using the scale at the bottom. **(C)** Distribution of conserved motifs in BnaSPX proteins. Different motifs are shown by different colors numbered 1–15. Some motifs are highlighted with different colored boxes with numbers. Lines represent protein regions without a detected motif.

Since the intron/exon organization and numbers are typical imprints of evolution within some gene families, we analyzed the gene structures of *BnaSPXs* by comparing the gDNA sequences with their corresponding coding sequences. The intron-exon structures were plotted along with the order of subfamily genes in phylogenetic tree (**Figure 2A**). The schematic structures revealed that each SPX gene coding sequence is disrupted by one or more introns (**Figure 2B**). SPX-EXS subfamily genes contain the largest number of introns, whereas SPX subfamily genes contain the fewest. The gene structures of most *BnaSPXs*, in terms of length and number of exons were similar to their homologous gene in *Arabidopsis*, especially for SPX, SPX-MFS, and SPX-RING subfamily genes. However, the genes in subfamily SPX-EXS exhibited diverse exon-intron gain/loss variations. By examining the exon-intron organization and paralogous pairs of SPX-EXS genes that were clustered together at the terminal branch of the phylogenetic tree, various exon-intron changes were identified. For instance, only one out of the four *BnaPHO1* genes, *BnaA1.PHO1*, shared the same exon-intron structure with *AtPHO1*, which contained 15 exons. The other three *BnaPHO1* genes (*BnaA7.PHO1, BnaC1.PHO1*, and *BnaC7.PHO1*) exhibited exon-intron gain/loss variations (**Figure 2B**), possibly due to a single intron loss or a gain event during the long evolution process.

**FIGURE 3:**
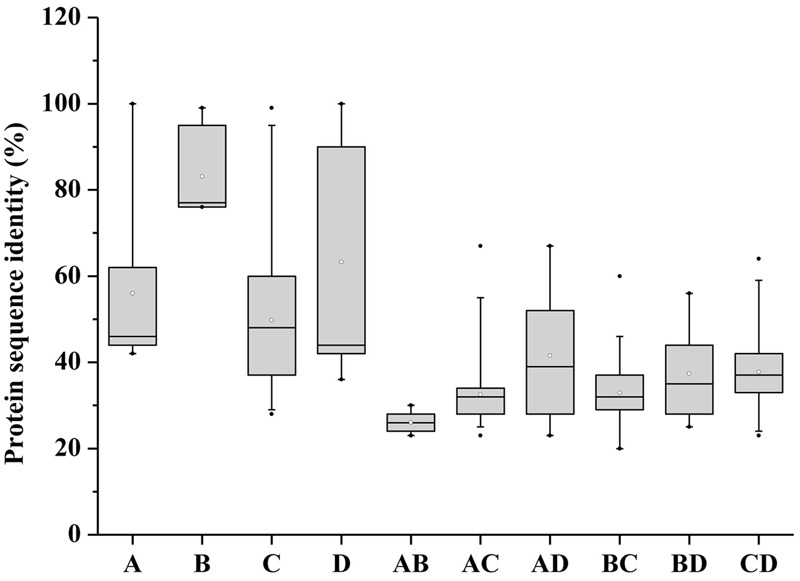
**Pairwise sequence identity of full-length BnaSPX proteins.** A, B, C, and D represent pairwise sequence identities of the SPX, SPX-MFS, SPX-EXS, and SPX-RING groups, respectively. AB, AC, and AD represent pairwise sequence identities between SPX and SPX-MFS, SPX and SPX-EXS, and SPX and SPX-RING proteins, respectively; BC and BD represent pairwise sequence identities between SPX-MFS and SPX-EXS and SPX-MFS and SPX-RING proteins, respectively; CD represents pairwise sequence identities between SPX-EXS and SPX-RING proteins. The box plot shows the median (black line), interquartile range (box), and maximum and minimum scores (whiskers) for each data set.

The conserved motifs of BnaSPX proteins were investigated further using MEME (**Figure 2C**), revealing a total of 15 conserved motifs (designated motifs 1–15). Motif 1, 7, and 12 specifying the conserved SPX domain indicated by the Pfam codes and WebLogo (**Supplementary Figure S2**) were present in almost all SPX family members. *BnaA6.PHO1;H3a, BnaC1.PHO1*, and *BnaC2.PHO1;H9* did not show motif 12, and SPX-MFS and SPX-RING subfamily members did not contain motif 1. The remaining motifs corresponded to the regions outside of the SPX domain region, which were distributed in specific groups in the phylogenetic tree. Proteins in the same group or subgroup contained similar motifs, whereas the motifs were divergent among different subfamilies. Conserved motifs 8, 10, and 11 specified the MFS domain and were found in all BnaSPX-MFS proteins. Motif 15 was only present in each member of the SPX-RING subfamily (**Figure 2C**), which indicated that this motif was specific to SPX-RING genes. Similarly, the most prominent feature of proteins in the SPX-EXS group was the EXS domain, which embraced at most seven motifs, and motifs 3 and 14 were shared by all members of the SPX-EXS subfamily. These results indicated that most motifs were distributed among specific groups, which correlated with their functional divergence. Pairwise comparisons of the 69 full-length BnaSPX protein sequences revealed that amino acid sequences in the SPX-EXS group were more diverse but well conserved in the SPX-MFS subfamily (**Figure 3**). Otherwise, the protein sequence identity within each group was higher than that between groups.

### RNA-Seq-Based Expression Profiling of *BnaSPX* Genes during Pi Deficiency Stress

**FIGURE 4:**
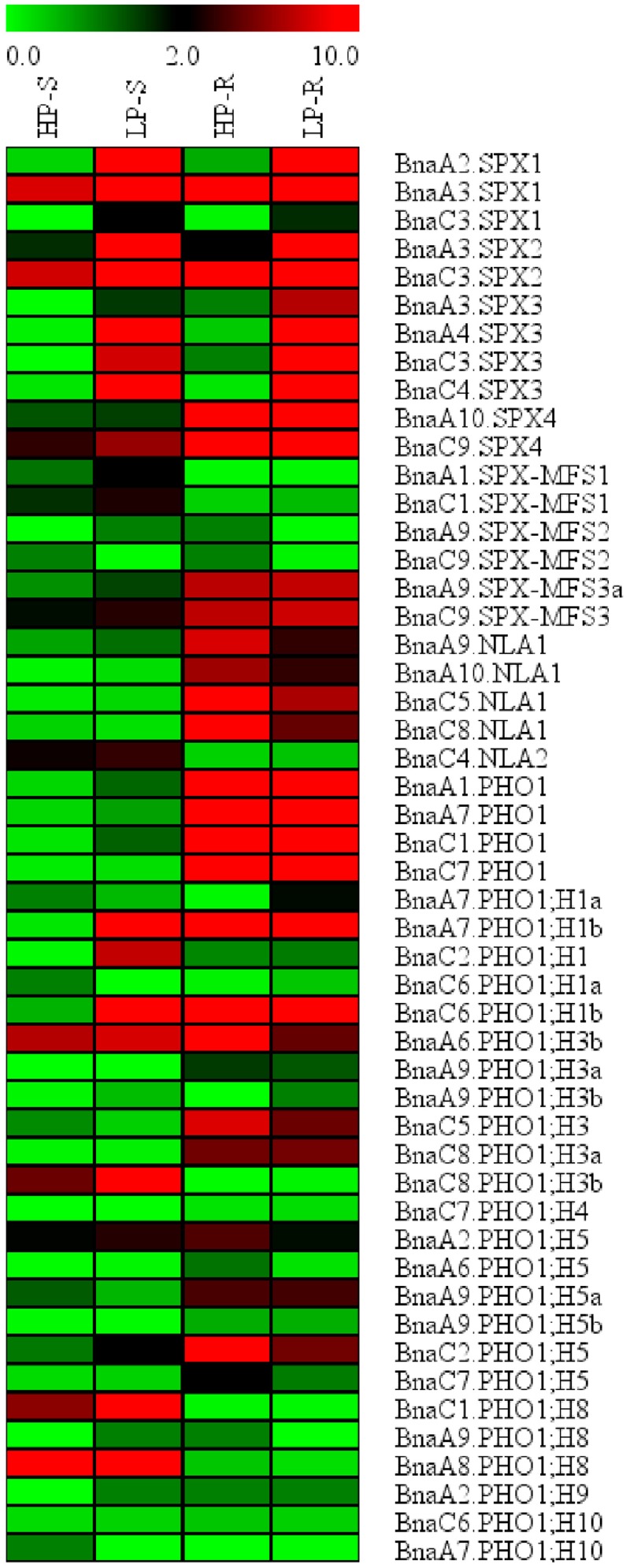
**Expression profile of *BnaSPX* genes in response to Pi stress.** Normalized gene expression values are shown in different colors that represent the relative levels of expression indicated on the scale bar. +P/-P indicated normal Pi or Pi starvation, respectively. S/R denotes shoot or root.

To identify potential *BnaSPX* genes involved in Pi starvation signaling, we analyzed the expression profiles of all SPX genes identified in *B. napus* under Pi stress using RNA-Seq analysis. A total of 50 *BnaSPX* genes were detected in the shoots and/or roots at the seedling stage (**Figure 4**). Among these *BnaSPX* genes, all SPX subfamily genes except *BnaSPX4s* were significantly up-regulated by Pi stress both in the shoot and root, especially for *BnaA2.SPX1, BnaA3.SPX1, BnaC3.SPX2, BnaA4.SPX3*, and *BnaC4.SPX3*, whereas *SPX-MFS1s* and *SPX-MFS3s* were moderately induced in response to Pi stress only in the shoot and not in the root. Four paralogous *BnaNLA1* genes were slightly down-regulated in the root whereas *BnaC4.NLA2* had no obvious change in either shoot or root when confronted with Pi stress. The expression levels of the 22 SPX-EXS subfamily genes were detected in response to Pi stress, among these, only *BnaPHO;H1* genes were induced with a twofold or great change in Pi stress condition (Supplementary Table S3). In contrast, two members of the *BnaPHO;H3* genes, *BnaA6.PHO1;H3b*, and *BnaC5.PHO1;H3*, were mildly suppressed in one of the examined tissues (Supplementary Table S3). The gene expression profiles revealed that two *BnaPHO;H5* genes, *BnaA2.PHO1;H5* and *BnaC2.PHO1;H5* showed diverse expression patterns in different tissues.

### qRT-PCR-Based Expression Profiles of *BnaSPX* Genes in Response to Pi Starvation

**FIGURE 5:**
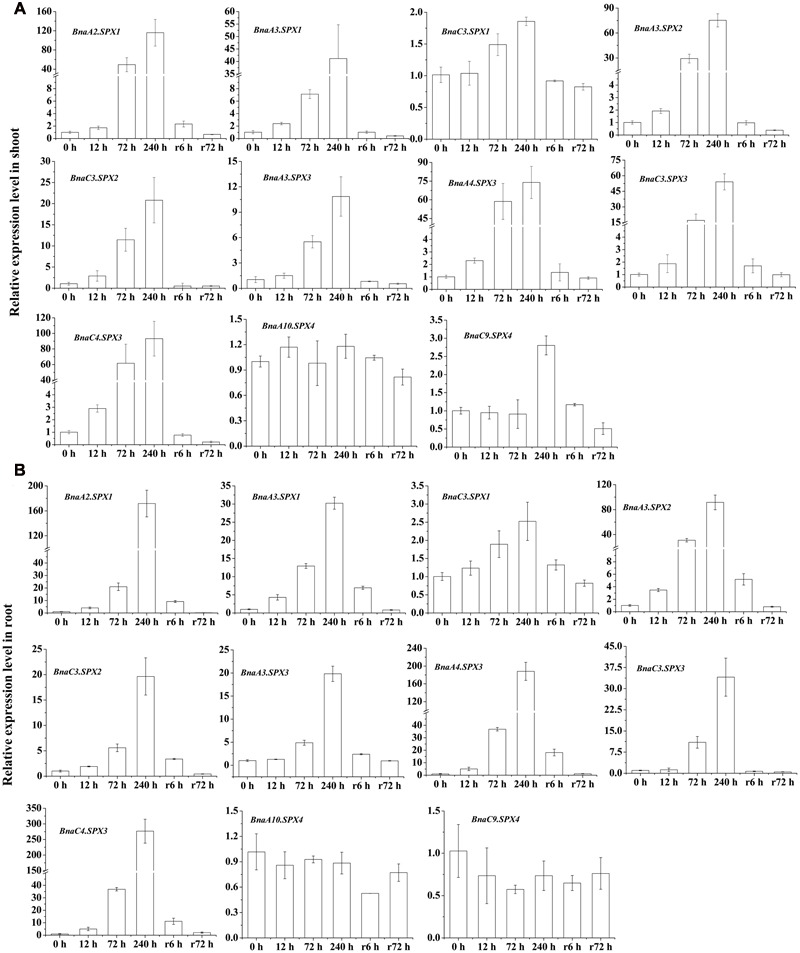
**Differential expression of *BnaSPX* genes in response to Pi starvation and Pi resupply.** Quantitative real-time RT-PCR was used to determine the transcript accumulation levels of *BnaSPXs* genes in shoots **(A)** and roots **(B)** of 15-day-old rapeseed plants hydroponically treated over additional time courses of Pi starvation and then Pi recovery. The data are the ratio of the signal at a given time to the signal at 0 h. Values represent mean ± standard deviation of three biological replicates.

To assess the validity and reliability of RNA-Seq data and obtain comprehensive insight into the expression profile of *BnaSPXs* during Pi starvation and Pi recovery, qRT-PCR was performed using gene-specific primers for 11 SPX subfamily genes. The expression profiles of all these chosen genes in 10 days were agreed with those obtained from the original RNA-Seq results (240 h), although the expression fold-change showed minimal differences (**Figure 5**). The results showed that 11 SPX subfamily genes, except for two *BnaSPX4* genes, were up-regulated at most of the time points after Pi starvation treatment. Overall, the PSI expression of most *BnaSPX* genes was observed within 12 h and peaked at 240 h. When the Pi-deprived plants were retransferred into sufficient Pi conditions, the elevated *BnaSPXs* transcripts were rapidly suppressed within 6 h and recovered to a normal level after 72 h of Pi-resupply. These results indicated that *BnaSPXs* were induced by Pi starvation continually and reversibly. We also found that some paralogs shared different induction sensitivity and intensity in response to Pi deficiency stress. Taking *BnaSPX1s* as an example, *BnaA2.SPX1* and *BnaA3.SPX1* had a high sensitivity to Pi stress by responding to Pi starvation early. Nevertheless, the Pi-starvation induction of *BnaC3.SPX1* was observed until 72 h. In contrast, four *BnaSPX3* paralogs showed a similar expression pattern to Pi deficiency; the transcript levels of all paralogous genes of *BnaSPX3* exhibited a rapid and significant increase triggered by Pi stress.

**FIGURE 6:**
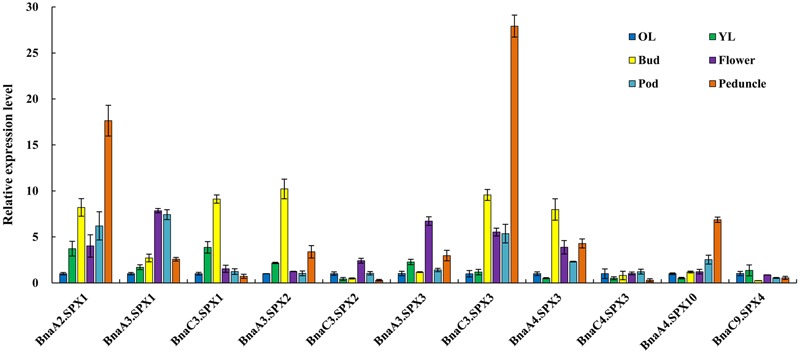
**Expression profiles of *BnaSPX* genes across different tissues under Pi deficiency.** The relative expression of *BnaSPXs* was analyzed by quantitative RT-PCR in old leaves (OL), young leaves (YL), buds, flowers, pods, and pod peduncles. The expression level of *BnaSPXs* in old leaves was set as 1. Values represent the mean ± SD of three biological replicates.

Because most *BnaSPXs* in the SPX subfamily are Pi-induced genes, qRT-PCR was performed to obtain an overview of the expression patterns of *BnaSPX* members in different tissues including old leaf (the lower third leaf), young leaf (the upper first leaf), bud, flower, pods, and pod peduncle under Pi-deficiency conditions (**Figure 6**). Two *BnaSPX1* paralogous genes (*BnaA2.SPX1* and *BnaA3.SPX1*), in addition to two *BnaSPX3* genes (*BnaC3.SPX3 and BnaA4.SPX3*), had predominant expression in one or more reproductive organs such as buds, flowers, pods, and pod peduncles while expressing these at low levels in vegetative tissues. *BnaA10.SPX4* had preferential expression in the peduncles, whereas the *BnaC3.SPX2, BnaC4.SPX3*, and *BnaC9.SPX4* genes were expressed at low levels in all tested tissues

### *Cis*-element Identification in the Promoter of SPX Family Genes

**FIGURE 7:**
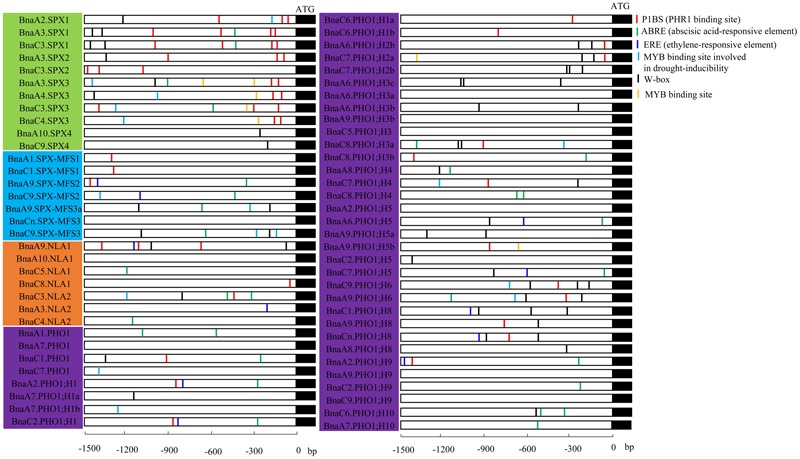
***Cis-*element analysis of the promoter regions of *BnaSPX* genes.** The micro-segments in different colors were the putative *cis*-element sequence. The description of the six *cis-*elements were in brackets. Four SPX subfamilies, including SPX, SPX-EXS, SPX-MFS, and SPX-RING, were distinguished by different background colors.

*Cis*-regulatory elements play important roles in the global regulation of gene expression. Growing evidence indicates that genes with similar expression patterns may contain the same regulatory elements in their promoters. Many identified Pi-starvation induced genes like signal molecules AtIPS1/At4, miRNAs, PTs, and purple acid phosphatases (PAPs), were found to be regulated by PHR1, the central regulator in the Pi signaling. The *cis*-element P1BS (PHR1 binding site), which contained an imperfect palindromic 8-bp sequence (GNATATNC) and was a conserved *cis*-element responding to Pi stress, showed gathered in the promoter of these PSR genes (Wu et al., 2013). The *cis*-elements in 66 *BnaSPXs*, expect for *BnaA6.PHO1;H2a, BnaA9.SPX-MFS3*, and *BnaA9.PHO1;H3a* with incomplete or low-quality promoter sequences, were analyzed using the online software PlantCARE based the *B. napus* genome data. Various types of *cis*-acting elements, including stress response-, hormone response-, and development-related elements, were detected in the promoter regions of *BnaSPX* genes (**Figure 7**), and most *BnaSPX* genes contained more than one *cis-*element type in their promoter regions, suggesting that these *BnaSPX* genes may be involved in complex regulatory networks. P1BS, a conserved *cis*-element responding to Pi stress, was significantly enriched in the promoters of SPX subfamily genes, with the exception of *Bna.SPX4s*. Two or three copies of the P1BS element were present in the promoters of these genes, most of which were distributed in the proximal terminal end of the promoters. However, no or only one copy of the P1BS element was found in the promoters of the other three SPX subfamily genes, and most P1BS was distributed in the distal promoters. Interestingly, we found that the W-box element was widely distributed in the promoters of *BnaPHO1;H2s, BnaPHO1;H3s, BnaPHO1;H6s*, and *BnaPHO1;H8s*, indicating that these genes may be regulated by WRKY transcription factors.

### Functional Divergence of Paralogous *BnaSPX* Genes in Transgenic *Arabidopsis*

**FIGURE 8:**
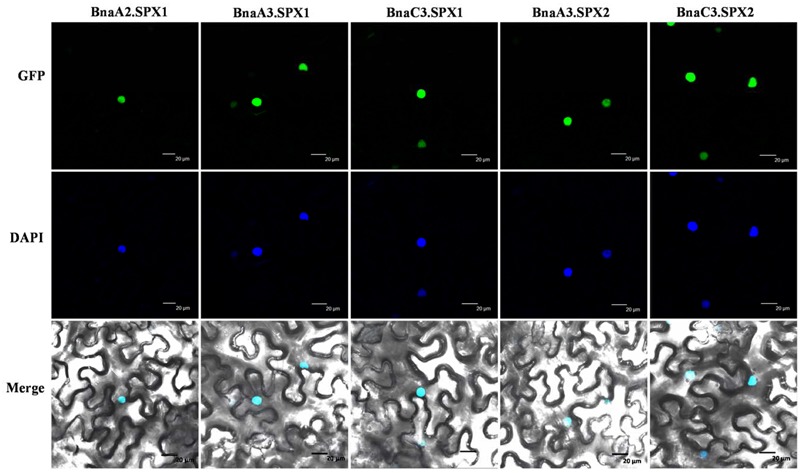
**Subcellular localization of BnaSPXs-GFP.** Confocal images were taken from *Nicotiana benthamiana* leaf epidermal cells. The positions of the nuclei are shown by DAPI staining. Bar = 20 μm

The paralogous BnaSPX proteins were highly similar to each other, with an amino acid identity/similarity ranging from 87/89 to 99/99% (**Supplementary Figure S3**). To further investigate the putative functional divergence among the paralogous SPX genes, we first determined the subcellular localization of selected BnaSPX1s and BnaSPX2s (**Figure 8**). BnaSPXs- green fluorescent protein (GFP) fusion proteins driven by a 35S promoter were transiently expressed in *N. benthamiana* leaves, and all the BnaSPXs-GFP fusion proteins were exclusively colocalized with the site of DAPI staining, indicating that all tested BnaSPX1s were localized in the nucleus.

**FIGURE 9:**
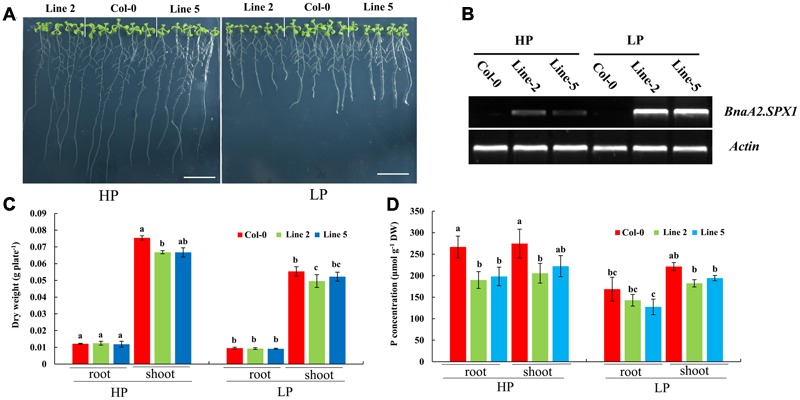
**Physiological effects and the influence on the Pi concentration of altering SPX1 activity in *Arabidopsis*. (A)** Phenotype of wild-type (Col-0) and transgenic lines (Line-2 and Line-5) with *BnaA2.SPX1* grown for 9 days under HP (1000 μM Pi) and LP (50 μM Pi) conditions; bar, 2 cm. **(B)** Semi-qRT-PCR analysis of the expression level of *BnaA2.SPX1* in Col-0 and transgenic lines (Lines 2 and 5) under HP and LP conditions. *Atactin7*, was used as the internal control. All the experiments were repeated two times with identical results. **(C,D)** Dry weight **(C)** and total P concentration **(D)** in shoots and roots of WT and two transgenic lines grown under two Pi conditions. Different letters on the bar represent statistically difference at *P* < 0.05.

**FIGURE 10:**
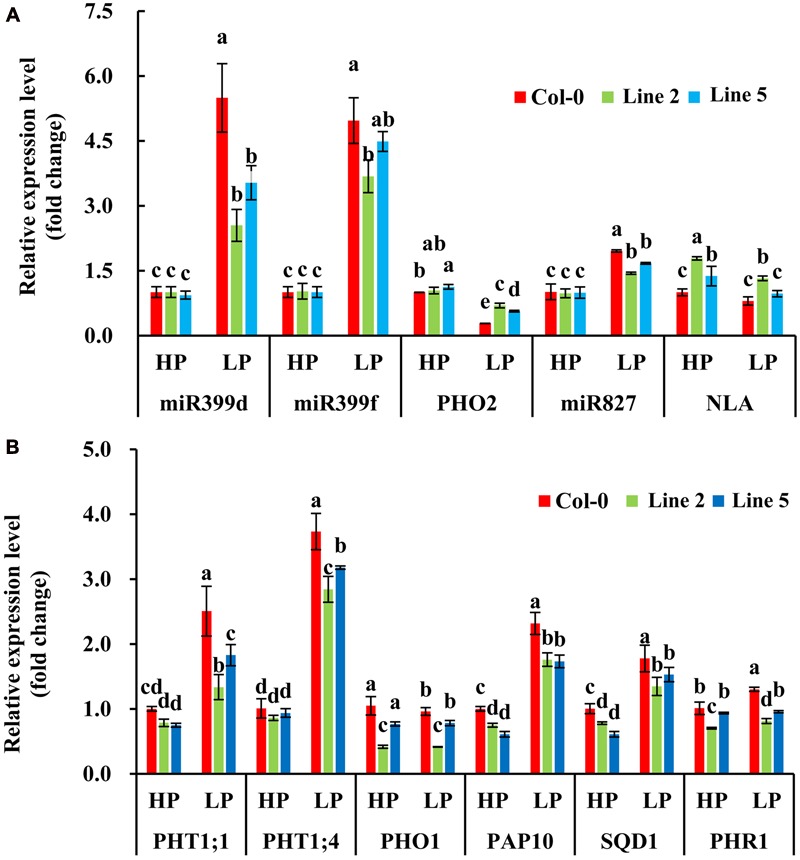
**Quantitative real time PCR analysis of the transcript accumulation levels of the components in Pi homeostasis signaling in seedlings of Col-0 and transgenic plants under two Pi conditions **(A,B)**.** Values represent mean ± standard deviation of three biological replicates, and different letters on the bars represent statistically significant difference at *P* < 0.05.

Then, we examined whether the two nuclear SPX1 proteins BnaA2.SPX1 and BnaC3.SPX1, which have a relatively low protein similarity (87%), were equally involved in Pi homeostasis. Transgenic *Arabidopsis* harboring the gDNA of *BnaA2.SPX1* and *BnaC3.SPX1* under the control of their native promoter were generated and evaluated for changes in dry weight and total P content under HP (1000 μM) and LP (50 μM) conditions. Interestingly, transgenic lines expressing the *BnaC3.SPX1* gene had no obvious phenotypic differences in comparison to the wild type despite Pi status (**Supplementary Figure S4**), whereas transgenic lines expressing the *BnaA2.SPX1* gene displayed retarded growth and showed more sensitivity to Pi deficiency compared to wild-type Col-0, especially during Pi deficiency (**Figures 9A,B**), The two transgenic lines (A2.SPX1-2 and A2.SPX1-5) showed reduced plant dry weight and total P concentration relative to wild-type Col-0 (**Figures 9C,D**). Furthermore, transgenic plant harboring p*BnaA2.SPX1::BnaA2.SPX1* altered the expression of a subset of Phosphate Stress Response (PSR) genes (**Figure 10B**). The tested genes, including phosphate transporters (PTs), like PHT1;1, PHT1;4 and other PSR genes, SQD1, and PAP10, showed decreased expression in overexpressing lines relative to the Col-0 in Pi deficiency condition, miRNAs like miRNA399, miRNA 827 were also suppressed whereas their target gene PHO2 and NLA were slightly induced in the transgenic plants (**Figure 10A**).

## Discussion

In the present study, we performed a comprehensive search for SPX domain-containing genes throughout the *B. napus* genome, and a total of 69 full-length SPX genes were identified. These genes were divided into four distinct subfamilies based on the domain organization and phylogenetic analysis, which is highly consistent with the results from *Arabidopsis* and rice (Secco et al., 2012). We also found that different gene members from the SPX family were highly preserved after the formation of the tetraploid, showing that the genome structure has been stable, i.e., a very low rate of gene loss, which has often been proposed to be widespread during the early stage of a neo-polyploid, thus contributing to rapid diversification of new plants (Jiao et al., 2011; Liu et al., 2014). In fact, for some artificial/synthetic tetraploids, chromosomal DNA and gene loss rates can be up to 15% during the first generations (Ozkan et al., 2002). Therefore, our findings show that an appreciable span of genome stability should occur for polyploids. Some polyploids may have very unstable genomes, whereas others may be stable. The *B. napus* genome seems to be on the more stable side.

Compared with the number of SPX members in *A. thaliana* and *O. sativa*, which contain 20 and 14 SPX genes, respectively (Secco et al., 2012), the number of SPX genes in rapeseed is remarkably high at 69 members. Copy number expansion of the SPX family in the *B. napus* genome has primarily occurred through genome duplication events (Chalhoub et al., 2014). Polyploidy or whole-genome duplication (WGD) has occurred multiple times throughout the evolutionary history of plants, providing raw genetic material for biological evolution and phenotypic innovation that helps organisms adapt to new and changing environments (Bowers et al., 2003). Gene structure analyses indicated that most *BnaSPX* genes in the same subfamily shared similar exon-intron organization, particularly within the SPX-RING group, where each member has precisely six exons. Considering the SPX-RING gene structures reported in other species, all genes in *Arabidopsis* and rice had six exons. The generally well-conserved exon structure in SPX-RING genes across different species highlights the possibility of conserved gene function and strict regulation of these genes. In contrast, the genes in the SPX-EXS subgroup exhibited diverse exon-intron gain/loss variations resulting from the integration or realignment of gene fragments in the SPX-EXS subgroup genes, which may play important roles for gene functional diversity (Xu et al., 2012).

### Exploring the Functions of *BnaSPX1* Paralogs in Response to Pi Stress

The functions of most SPX subfamily genes have been well characterized in *Arabidopsis* and rice (Lv et al., 2014; Puga et al., 2014; Shi et al., 2014). In this study, we analyzed the gene expression patterns of *BnaSPXs* in responding to Pi stress by RNA-Seq data and qRT-PCR analysis, both results indicated that *BnaSPXs* in SPX subfamily were significantly involved in the Pi signaling pathway, which were in consistent with our previous study that *BnSPX3;1* and *BnSPX3;2* (here designated as *BnaA3.SPX3* and *BnaC3.SPX3*, respectively) were P-specific induced genes and the induction were rapid and durative during Pi starvation, and reversible upon resupply of Pi (Yang et al., 2011). Unlike *BnaSPX3s*, which were Pi-starvation-specific induced genes, *BnaSPX1* members expressed at a low level in normal Pi condition but induced significantly in Pi deficiency condition. We selected two *BnaSPX1s* as the candidates for further functional analysis in *Arabidopsis* by introducing *BnaSPX1s* into *Arabidopsis* under their native promoters. The expression of *BnaA2.SPX1* in *Arabidopsis* decreased the transcript level of a subset of PSR genes (**Figure 10**), including PTs, SQD1, and PAP10, and miRNAs implying that *BnaA2.SPX1* was a negative regulator in the Pi signaling network. Consistent results were obtained which indicated the *Arabidopsis* SPX1 located in nuclear and acted as a direct Pi-dependent inhibitor of PHR1 by inhibiting the binding to its PSI targets via P1BS motif (Puga et al., 2014). But contradictory results found that transgenic plant with 35::AtSPX1 vector in *Arabidopsis* enhances the transcription of several PSI (e.g., AtACP5, AtPAP2, and AtRNS1), indicating a positive regulatory role for AtSPX1 (Duan et al., 2008). Another study in rice showed that OsSPX1 is a negative regulator of OsPHR2 (the homolog of AtPHR1) and was involved in the feedback of Pi-signaling network in roots that is defined by OsPHR2 and OsPHO2 (Wang et al., 2009). The molecular mechanisms of SPX regulation in Pi acquisition and translocation need to be further clarified.

We identified 3 *BnaSPX1* genes (*BnaA2.SPX1, BnaA3.SPX1*, and *BnaC3.SPX1*) in *B. napus* by checking genes within the same taxonomic group on the phylogenetic tree. The fate of the paralogs resulted from whole genome duplication events has attracted substantial interest with respect to evolutionary novelty, species fitness and diversity. Previous studies have discussed three alternative outcomes of gene duplication: neofunctionalization, subfunctionalization, and non-functionalization (Lynch and Conery, 2000; Li et al., 2005). In the neofunctionalization model, one duplicate copy (paralog) accumulates beneficial mutations and acquires a new function, whereas the other duplicate copy retains the original gene function. In the subfunctionalization model, each paralog partitions the ancestral gene function (Li et al., 2005). The process of non-functionalization can occur when a redundant gene degenerates to a pseudogene or is lost from the genome due to chromosomal remodeling, locus deletion or point mutation (Gu, 2003). Notably, the biochemical and functional diversity of paralogs may enable polyploids to better adapt to unfavorable environment than their diploid parents. Divergent expression patterns responding to Pi stress were detected among *BnaSPX1* paralogs. *BnaA2.SPX1* and *BnaA3.SPX1* genes were rapidly and significantly up-regulated by Pi stress, whereas the induction of *BnaC3.SPX1* was delayed and slight unless long-term Pi starvation occurred (**Figure 5**). The expression profiles of *BnaSPX1* genes across different tissues under Pi deficiency indicated that *BnaA2.SPX1* and *BnaA3.SPX1* had predominant expression in reproductive organs such as flowers, pod and pod peduncles, whereas *BnaC3.SPX1* was expressed at low levels in the above-mentioned tissues (**Figure 6**). Although all the paralogous BnaSPX1 proteins are located in nuclear (**Figure 8**), surprisingly, only *BnaA2.SPX1* negatively regulated Pi signaling; another paralog, *BnaC3.SPX1*, was not significantly involved in Pi homeostasis (**Figures 9** and **10**; **Supplementary Figure S4**). First, TF binding sites (TFBSs) or *cis*-elements, upstream of target genes can regulate gene expression and then alter gene function. Based on the analysis of *cis*-regulatory elements of the *B. napus* genome, there seems to be no correlation between *cis*-regulatory elements and expression bias among paralogous *BnaSPX1s* (**Figure 7**). In fact, all *BnaSPX1* paralogs maintained the conserved P1BS elements with similar position and number in their promoters. Whether other *cis*-regulons exist in the promoters of these two paralogs to confer different expression bias remains to be elucidated. We also considered another possible explanation, i.e., whether epigenetic modifications such as DNA methylation and histone modification were responsible for the different Pi starvation responses of *BnaSPX1* paralogs. It was shown that an adverse environment can induce genome-wide changes in DNA methylation status, specifically in stress-related genes in *B. napus* (Gao et al., 2014; Verkest et al., 2015). As such, epigenetic modification analysis between these two genes will be performed in the future.

## Conclusion

In this study, we identified 69 *SPX* genes from the *B. napus* genome and established their classification and phylogeny using phylogenetic, gene structure, and conserved protein motif analyses. Expression profiles of *BnaSPXs* in response to Pi stress obtained from RNA-Seq data and qRT-PCR analysis indicated that SPX subfamily genes broadly participated in the Pi signaling pathway. Characterization of *Bna.SPX1* paralogs revealed their functional divergence during long-term evolution. This study provides further functional characterization of the *SPX* gene family in *B. napus* and provides a basis for exploiting candidate genes for genetic engineering of P efficiency in *B. napus*.

## Author Contributions

FX designed the study and provide guidance on the whole study. HD carried out the bioinformatic analysis, plant materials culture, qRT-PCR analysis, and drafted the manuscript. CY took parted in the plant materials culture and bioinformatic analysis. LS and GD provided the value comments and revised the grammar of the manuscript. All authors read and approved the final manuscript.

## Conflict of Interest Statement

The authors declare that the research was conducted in the absence of any commercial or financial relationships that could be construed as a potential conflict of interest.

## Footnotes

1 http://www.genoscope.cns.fr/brassicanapus/

2 http://smart.embl-heidelberg.de/

3 http://www.ncbi.nlm.nih.gov/Structure/cdd/wrpsb.cgi

4 http://www.ebi.ac.uk/Tools/pfa/iprscan/

5 http://www.expasy.org/tools/

6 http://web.expasy.org/protparam/

7 http://www.genscript.com/wolf-psort.html

8 http://www.cbs.dtu.dk/services/TargetP/

9 http://brassicadb.org/brad/searchSyntenytPCK.php

10 http://alternate.meme-suite.org/

11 http://gsds.cbi.pku.edu.cn/

12 http://brassicadb.org/cgi-bin/gbrowse/B.napus_chromosome/

13 http://bioinformatics.psb.ugent.be/webtools/plantcare/html/

## Supplementary Material

FIGURE S1 **Distribution of *BnaSPX* members on *B. napus* chromosomes.** chromosome number was defined as A01 to C09 on the top of each chromosome. The size of each chromosome can be estimated from the bottom number (Mb). The 67 *BnaSPXs* were mapped to the 18 *B. napus* chromosomes, except A05. The number following each gene represents the exact chromosomal location that was available in the *B. napus* database.

FIGURE S2 **WebLogo plot of consensus motifs in SPX domain of *BnaSPX* gene family**.

FIGURE S3 **Identity/similarity matrix of SPX subfamily proteins.** Amino acid identity and similarity are indicated by the first and second number and the amino acid similarity and identity of paralogous SPX genes are indicated as red.

FIGURE S4 **Physiological effects of altering *BnaC3.SPX1* activity in *Arabidopsis*. (A)** The expression level of *BnaC3.SPX1* in Col-0 and transgenic lines (Line 1 and Line 2) under HP (1000 μM Pi) and LP (50 μM Pi) conditions by semi-qRT-PCR analysis. AtACTIN7 was used as the internal control. **(B)** Phenotype of wild-type (Col-0) and transgenic lines (Line-1 and Line-2) with *BnaC3.SPX1* grown for 11 days under HP and LP conditions; bar, 2 cm.

## Abbreviations

AS: acetosyringone
BLAST: basic local alignment search tool
DAPI: 4′, 6-diamino-phenylindole
FPKM: fragment per Kb of exon model per million reads
GRAVY: the grand average of hydropathy
MEME: multiple expectation maximization for Motif Elicitation
MES: 2-(*N*-morpholino)ethanesulfonic acid hydrate
Pi: inorganic phosphate
PSI: Pi starvation-induced genes
qRT-PCR: quantitative real time PCR
