# Supplementary figures and images for: Genome-Wide Identification and Characterization of SPX Domain-Containing Members and Their Responses to Phosphate Deficiency in Brassica napus
Source: Front Plant Sci. 2017 Jan 25;8:35. doi: 10.3389/fpls.2017.00035 (PMC5263162; doi:10.3389/fpls.2017.00035)

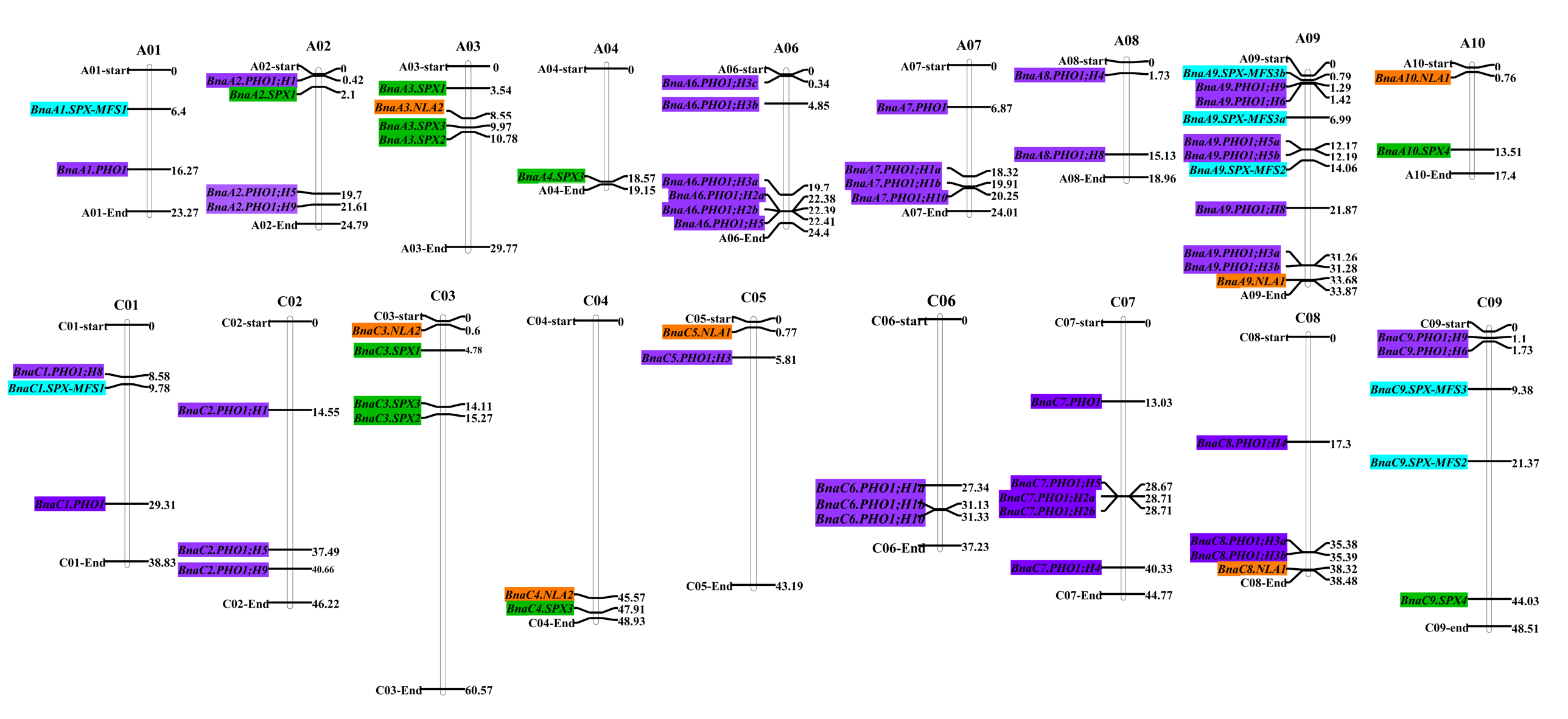

Supplement: FIGURE S1 — Distribution of BnaSPX members on B. napus chromosomes. chromosome number was defined as A01 to C09 on the top of each chromosome. The size of each chromosome can be estimated from the bottom number (Mb). The 67 BnaSPXs were mapped to the 18 B. napus chromosomes, except A05. The number following each gene represents the exact chromosomal location that was available in the B. napus database. [file Image_1.JPEG]

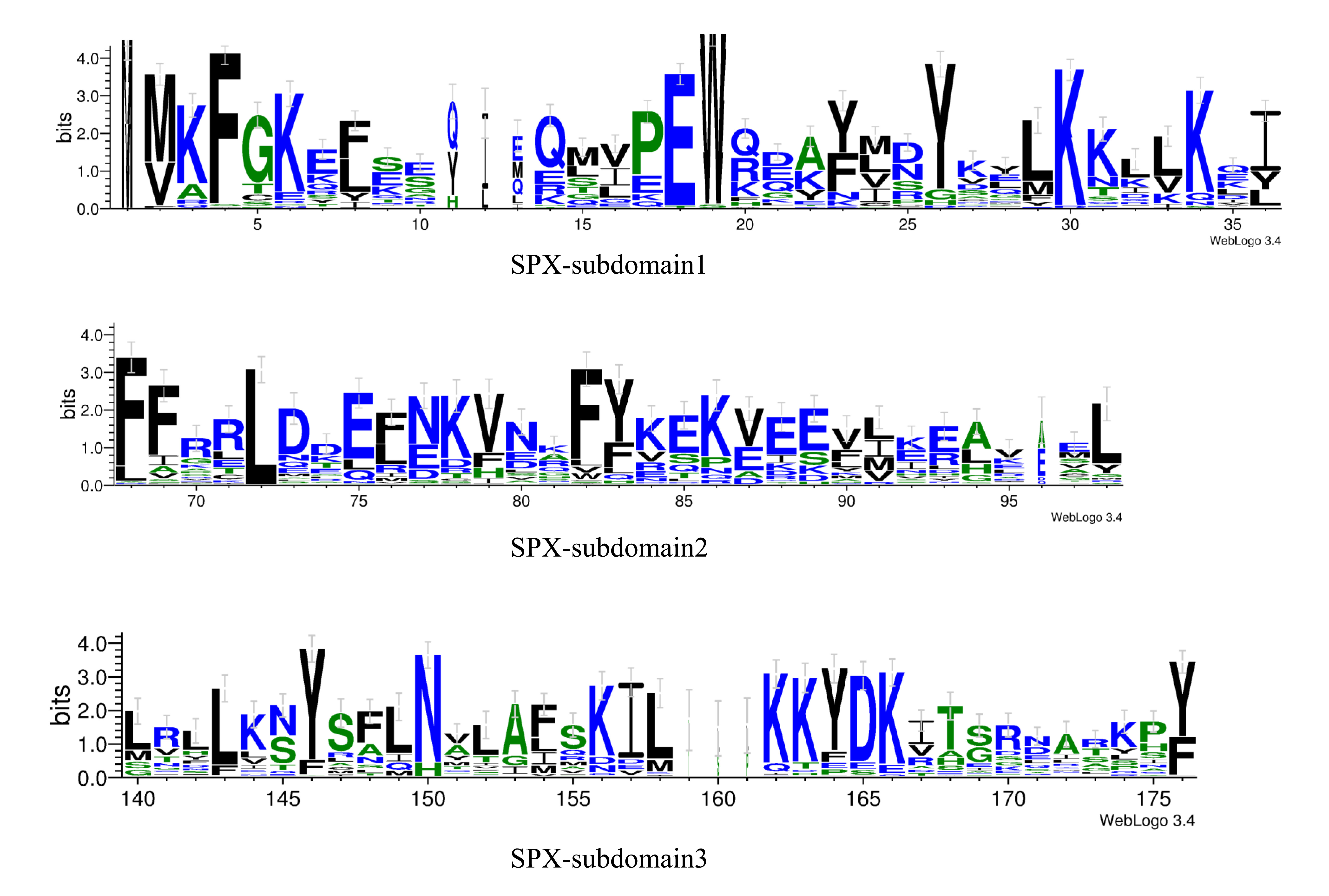

Supplement: FIGURE S2 — WebLogo plot of consensus motifs in SPX domain of BnaSPX gene family. [file Image_2.JPEG]

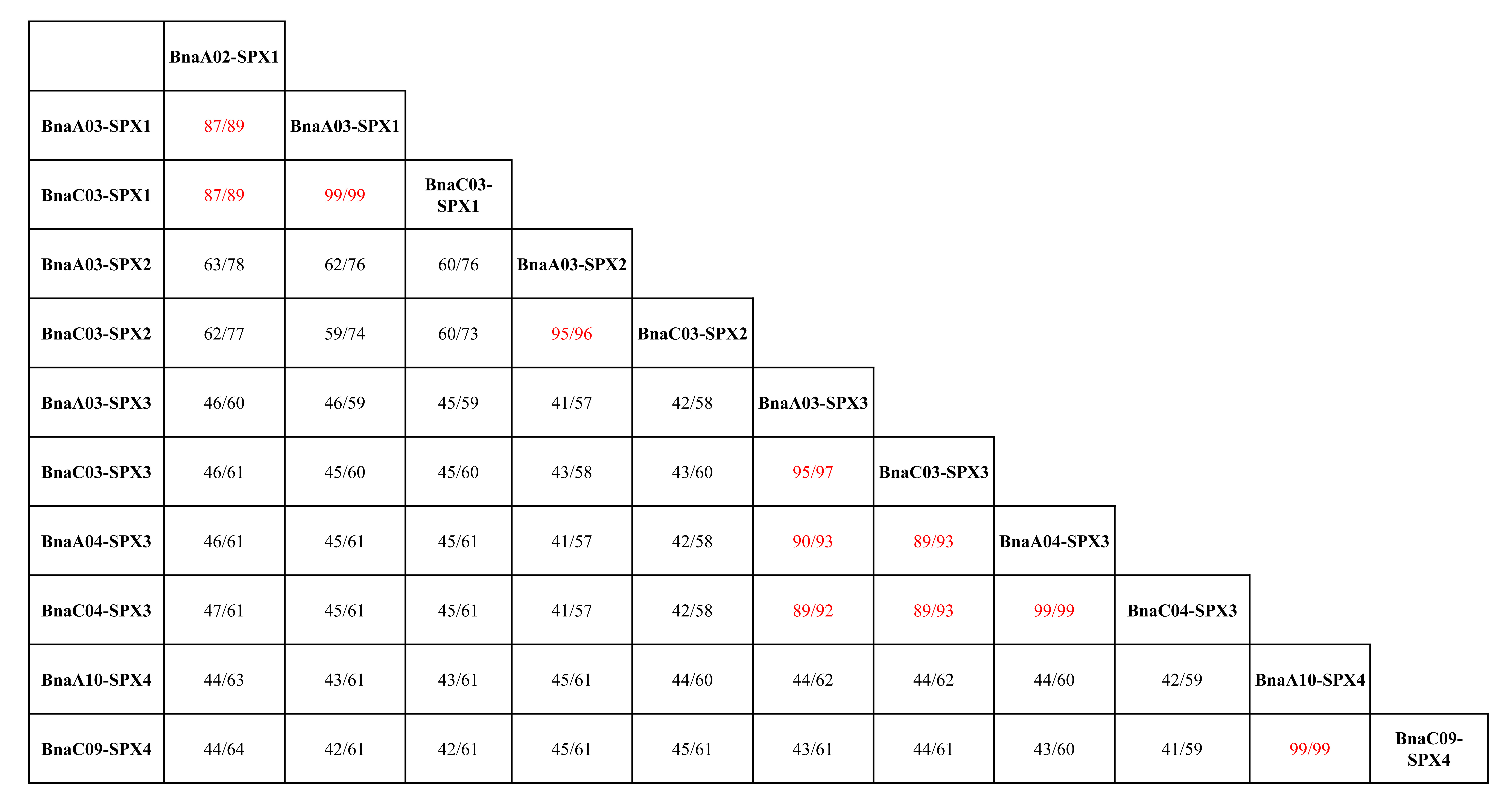

Supplement: FIGURE S3 — Identity/similarity matrix of SPX subfamily proteins. Amino acid identity and similarity are indicated by the first and second number and the amino acid similarity and identity of paralogous SPX genes are indicated as red. [file Image_3.JPEG]

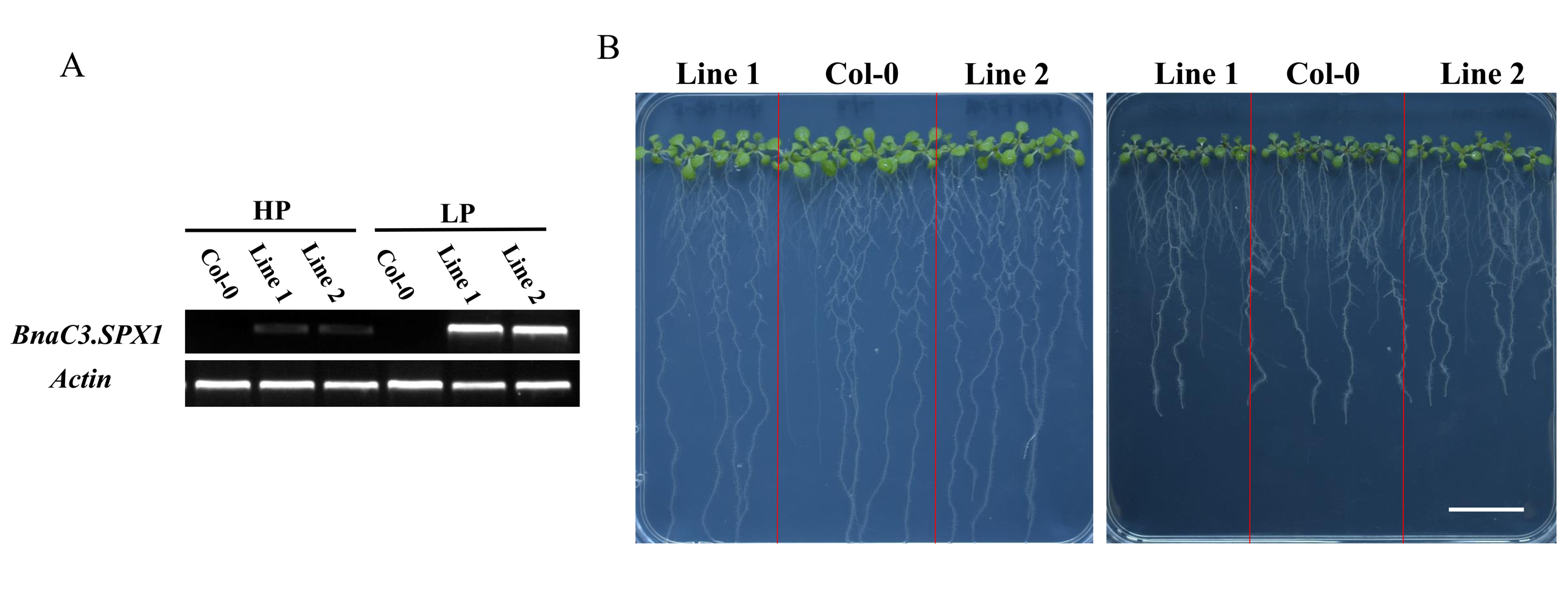

Supplement: FIGURE S4 — Physiological effects of altering BnaC3.SPX1 activity in Arabidopsis. (A) The expression level of BnaC3.SPX1 in Col-0 and transgenic lines (Line 1 and Line 2) under HP (1000 μM Pi) and LP (50 μM Pi) conditions by semi-qRT-PCR analysis. AtACTIN7 was used as the internal control. (B) Phenotype of wild-type (Col-0) and transgenic lines (Line-1 and Line-2) with BnaC3.SPX1 grown for 11 days under HP and LP conditions; bar, 2 cm. [file Image_4.JPEG]
