# Supplementary material for: Genome-Wide Identification and Characterization of SPX Domain-Containing Members and Their Responses to Phosphate Deficiency in Brassica napus
Source: Front Plant Sci. 2017 Jan 25;8:35. doi: 10.3389/fpls.2017.00035 (PMC5263162; doi:10.3389/fpls.2017.00035)
Supplement: Supplementary file 5 [file Table_1.DOCX]

**Supplementary Table 1. *SPX* genes in *B. napus* genome and their sequence characteristics and subcellular location prediction.**

| **Gene ID** | **Gene name** | **NO. of exon** | **Protein properties** | | | **Subcellular location** | | **GRAVY^c^** | **Best hit** | **Group** |
| --- | --- | --- | --- | --- | --- | --- | --- | --- | --- | --- |
|  |  |  | **Amino acid** | **MW(kDa)** | **PI** | **Targetp^a^** | **Wolf Psort^b^** |  |  |  |
| BnaA02g04730D | *BnaA2.SPX1* | 3 | 265 | 31.23 | 7.7 | other | nucl | -0.586 | *AtSPX1* AT5G20150 | **SPX** |
| BnaA03g07930D | *BnaA3.SPX1* | 2 | 255 | 29.8 | 6.88 | other | nucl | -0.511 |  |  |
| BnaC03g10000D | *BnaC3.SPX1* | 2 | 255 | 29.77 | 6.87 | other | nucl | -0.488 |  |  |
| BnaA03g22680D | *BnaA3.SPX2* | 3 | 281 | 32.41 | 5.19 | other | nucl | -0.641 | *AtSPX2* AT2G26660 |  |
| BnaC03g26700D | *BnaC3.SPX2* | 3 | 281 | 32.32 | 5.28 | other | nucl | -0.625 |  |  |
| BnaA03g21050D | *BnaA3.SPX3* | 3 | 246 | 28.54 | 6.26 | other | cyto | -0.351 | *AtSPX3* AT3G45130 |  |
| BnaA04g26060D | *BnaA4.SPX3* | 3 | 262 | 30.32 | 6.87 | other | nucl | -0.316 |  |  |
| BnaC03g25110D | *BnaC3.SPX3* | 3 | 239 | 27.61 | 6.37 | other | cyto | -0.411 |  |  |
| BnaC04g50120D | *BnaC4.SPX3* | 3 | 247 | 28.56 | 8.51 | other | cyto | -0.373 |  |  |
| BnaA10g18780D | *BnaA10.SPX4* | 3 | 304 | 34.86 | 4.91 | other | nucl | -0.743 | *AtSPX4* AT5G15330 |  |
| BnaC09g42510D | *BnaC9.SPX4* | 3 | 303 | 34.64 | 4.95 | other | nucl | -0.713 |  |  |
| BnaA01g12800D | *BnaA1.SPX-MFS1* | 9 | 600 | 78.38 | 6.02 | M | PM | 0.204 | *AtSPX-MFS1* At4g22990 | **SPX-MFS** |
| BnaC01g14580D | *BnaC1.SPX-MFS1* | 9 | 695 | 77.77 | 6.36 | M | PM | 0.223 |  |  |
| BnaA09g21340D | *BnaA9.SPX-MFS2* | 10 | 704 | 78.9 | 5.44 | other | PM | 0.198 | *AtSPX-MFS2* At4g11810 |  |
| BnaC09g23750D | *BnaC9.SPX-MFS2* | 10 | 704 | 78.88 | 5.44 | other | PM | 0.201 |  |  |
| BnaA09g12960D | *BnaA9.SPX-MFS3a* | 9 | 697 | 78.09 | 6.77 | M | nucl | 0.164 | *AtSPX-MFS3* At1g63010 |  |
| BnaA09g53000D | *BnaA9.SPX-MFS3b* | 10 | 702 | 78.53 | 5.92 | other | nucl | 0.161 |  |  |
| BnaCnng51450D | *BnaCn.SPX-MFS3* | 10 | 702 | 78.56 | 5.82 | other | nucl | 0.166 |  |  |
| BnaC09g12880D | *BnaC9.SPX-MFS3* | 9 | 694 | 77.8 | 6.77 | M | nucl | 0.175 |  |  |
| BnaA09g51130D | *BnaA9.NLA1* | 6 | 331 | 37.93 | 8.49 | other | nucl | -0.386 | *AtNLA1* At1g02860 | **SPX-RING** |
| BnaA10g01450D | *BnaA10.NLA1* | 6 | 333 | 38.24 | 8.48 | other | nucl | -0.421 |  |  |
| BnaC05g01480D | *BnaC5.NLA1* | 6 | 332 | 38.13 | 8.83 | other | nucl | -0.413 |  |  |
| BnaC08g45940D | *BnaC8.NLA1* | 6 | 330 | 37.92 | 8.65 | other | nucl | -0.395 |  |  |
| BnaC03g72250D | *BnaC3.NLA2* | 6 | 333 | 38.76 | 8.52 | other | cyto | -0.274 | *AtNLA2* At2g38920 |  |
| BnaA03g18240D | *BnaA3.NLA2* | 6 | 333 | 38.58 | 8.61 | other | cyto | -0.253 |  |  |
| BnaC04g46040D | *BnaC4.NLA2* | 6 | 333 | 38.8 | 8.62 | other | cyto | -0.266 |  |  |
| BnaA01g23840D | *BnaA1.PHO1* | 15 | 762 | 88.91 | 9.48 | other | PM | 0.03 | *AtPHO1* At3g23430 | **SPX-EXS** |
| BnaA07g06530D | *BnaA7.PHO1* | 16 | 812 | 93.5 | 9.46 | other | PM | -0.114 |  |  |
| BnaC01g30800D | *BnaC1.PHO1* | 13 | 598 | 68.53 | 9.47 | other | PM | 0.086 |  |  |
| BnaC07g08080D | *BnaC7.PHO1* | 13 | 564 | 66.09 | 9.09 | other | PM | 0.128 |  |  |
| BnaA02g35630D | *BnaA2.PHO1;H1* | 12 | 770 | 89.37 | 9.01 | other | PM | -0.189 | *AtPHO1;H1* At1g68740 |  |
| BnaA07g24450D | *BnaA7.PHO1;H1a* | 13 | 798 | 92.68 | 8.95 | other | PM | -0.18 |  |  |
| BnaA07g27390D | *BnaA7.PHO1;H1b* | 12 | 770 | 89.24 | 9.14 | other | PM | -0.138 |  |  |
| BnaC02g18430D | *BnaC2.PHO1;H1* | 12 | 770 | 89.18 | 9.03 | other | PM | -0.177 |  |  |
| BnaC06g25630D | *BnaC6.PHO1;H1a* | 12 | 771 | 89.58 | 9.03 | other | PM | -0.16 |  |  |
| BnaC06g30370D | *BnaC6.PHO1;H1b* | 12 | 770 | 89.29 | 9.16 | other | PM | -0.147 |  |  |
| BnaA06g33750D | *BnaA6.PHO1;H2a* | 11 | 635 | 73 | 9.54 | other | PM | -0.211 | *AtPHO1;H2* At2g03260 |  |
| BnaA06g33780D | *BnaA6.PHO1;H2b* | 13 | 800 | 92.91 | 9.47 | other | PM | -0.162 |  |  |
| BnaC07g22190D | *BnaC7.PHO1;H2a* | 14 | 760 | 88.43 | 9.44 | other | PM | -0.097 |  |  |
| BnaC07g22200D | *BnaC7.PHO1;H2b* | 13 | 798 | 92.22 | 9.43 | other | PM | -0.166 |  |  |
| BnaA06g38130D | *BnaA6.PHO1;H3c* | 13 | 742 | 86.12 | 8.83 | other | PM | -0.262 | *AtPHO1;H3* At1g14040 |  |
| BnaA06g38150D | *BnaA6.PHO1;H3a* | 13 | 589 | 68.44 | 9.32 | S | PM | -0.076 |  |  |
| BnaA06g08940D | *BnaA6.PHO1;H3b* | 12 | 794 | 91.46 | 9.37 | other | PM | -0.18 |  |  |
| BnaA09g45870D | *BnaA9.PHO1;H3a* | 13 | 756 | 87.66 | 9.38 | other | PM | -0.076 |  |  |
| BnaA09g45890D | *BnaA9.PHO1;H3b* | 8 | 559 | 63.02 | 9.18 | other | PM | -0.229 |  |  |
| BnaC05g10300D | *BnaC5.PHO1;H3* | 13 | 825 | 95.14 | 9.48 | other | PM | -0.155 |  |  |
| BnaC08g39940D | *BnaC8.PHO1;H3a* | 13 | 762 | 88.16 | 9.32 | other | PM | -0.062 |  |  |
| BnaC08g39950D | *BnaC8.PHO1;H3b* | 13 | 780 | 90.04 | 9.25 | other | PM | -0.094 |  |  |
| BnaA08g30860D | *BnaA8.PHO1;H4* | 10 | 718 | 83.78 | 9.45 | other | PM | -0.096 | *AtPHO1;H4* At4g25350 |  |
| BnaC07g39540D | *BnaC7.PHO1;H4* | 11 | 726 | 84.73 | 9.48 | other | PM | -0.042 |  |  |
| BnaC08g12050D | *BnaC8.PHO1;H4* | 10 | 730 | 84.82 | 9.5 | other | PM | -0.072 |  |  |
| BnaA02g26710D | *BnaA2.PHO1;H5* | 9 | 817 | 94.88 | 8.85 | other | PM | -0.221 | *AtPHO1;H5* At2g03240 |  |
| BnaA06g33790D | *BnaA6.PHO1;H5* | 11 | 776 | 90.63 | 9.07 | other | PM | -0.193 |  |  |
| BnaA09g19200D | *BnaA9.PHO1;H5a* | 9 | 807 | 93.53 | 8.99 | other | PM | -0.172 |  |  |
| BnaA09g19210D | *BnaA9.PHO1;H5b* | 10 | 806 | 93.18 | 9.06 | other | PM | -0.17 |  |  |
| BnaC02g34860D | *BnaC2.PHO1;H5* | 9 | 817 | 94.84 | 9.04 | other | PM | -0.203 |  |  |
| BnaC07g22160D | *BnaC7.PHO1;H5* | 11 | 780 | 90.96 | 8.98 | other | PM | -0.202 |  |  |
| BnaC09g52350D | *BnaC9.PHO1;H6* | 12 | 783 | 90.31 | 9.3 | other | PM | -0.131 | *AtPHO1;H6* At2g03250 |  |
| BnaA09g53600D | *BnaA9.PHO1;H6* | 13 | 772 | 89.29 | 9.21 | other | PM | -0.08 |  |  |
| BnaC01g13200D | *BnaC1.PHO1;H8* | 11 | 747 | 86.98 | 9.31 | other | PM | -0.097 | *AtPHO1;H8* At1g35350 |  |
| BnaA09g29180D | *BnaA9.PHO1;H8* | 11 | 740 | 86.32 | 9.32 | other | PM | -0.015 |  |  |
| BnaCnng33210D | *BnaCn.PHO1;H8* | 11 | 738 | 86.21 | 9.29 | other | PM | -0.041 |  |  |
| BnaA08g19850D | *BnaA8.PHO1;H8* | 11 | 748 | 87.06 | 9.32 | other | PM | -0.096 |  |  |
| BnaA02g29650D | *BnaA2.PHO1;H9* | 13 | 787 | 91.09 | 9.32 | other | PM | -0.148 | *AtPHO1;H9* At3g29060 |  |
| BnaA09g02520D | *BnaA9.PHO1;H9* | 13 | 798 | 91.67 | 9.42 | other | PM | -0.193 |  |  |
| BnaC02g37670D | *BnaC2.PHO1;H9* | 14 | 649 | 75.61 | 9.45 | M | PM | -0.014 |  |  |
| BnaC09g01980D | *BnaC9.PHO1;H9* | 13 | 798 | 91.73 | 9.49 | other | PM | -0.191 |  |  |
| BnaC06g30650D | *BnaC6.PHO1;H10* | 12 | 783 | 91.09 | 8.74 | other | PM | -0.177 | *AtPHO1;H10* At1g69480 |  |
| BnaA07g27980D | *BnaA7.PHO1;H10* | 12 | 784 | 91.17 | 8.98 | other | PM | -0.194 |  |  |

a: TargetP 1.1 was employed to predict the subcellular location of eukaryotic proteins. M, Mitochondrion, i.e. the sequence contains mTP, a mitochondrial targeting peptide; S, Secretory pathway, i.e. the sequence contains SP, a signal peptide; other, any other location. b: nucl, PM and cyto predicted with Wolf Psort were the abbreviation of nucleus, plasma membrane and cytoplasm, respectively. c: GRAVY means grand average of hydropathy.
