# Supplementary material for: Genome-Wide Identification and Characterization of SPX Domain-Containing Members and Their Responses to Phosphate Deficiency in Brassica napus
Source: Front Plant Sci. 2017 Jan 25;8:35. doi: 10.3389/fpls.2017.00035 (PMC5263162; doi:10.3389/fpls.2017.00035)
Supplement: Supplementary file 6 [file Table_2.DOCX]

**Supplementary Table 2. Syntenic analysis of SPX-domain containing genes between *Brassica napus* and other *Brassicaceae* species.**

| **tPCK Chr^a^** | **Genomic block** | ***Arabidopsis*** | ***Brassica rapa*** | | | ***Brassica oleracea*** | | | ***Brassica napus* A genome** | | | ***Brassica napus* C genome** | | |
| --- | --- | --- | --- | --- | --- | --- | --- | --- | --- | --- | --- | --- | --- | --- |
|  |  |  | **LF^b^** | **MF1^c^** | **MF2^c^** | **LF^b^** | **MF1^c^** | **MF2^c^** | **LF^b^** | **MF1^c^** | **MF2^c^** | **LF^b^** | **MF1^c^** | **MF2^c^** |
| tPCK5 | R | AtSPX1 AT5G20150 | -- | Bra006543 | Bra020088 | Bol035841 | Bol034590 | Bol020103 | -- | BnaA3.SPX1 | BnaA2.SPX1 | -- | BnaC3.SPX1 | -- |
| tPCK4 | U | AtSPX2 AT2G26660 | -- | -- | Bra000539 | -- | -- | Bol032881 | -- | -- | BnaA3.SPX2 | -- | -- | BnaC3.SPX2 |
| tPCK3 | J | AtSPX3 AT3G45130 | -- | -- | -- | -- | -- | -- | -- | -- | -- | -- | -- | -- |
| tPCK5 | R | AtSPX4 AT5G15330 | Bra008702 | -- | -- | Bol030451 | -- | -- | BnaA10.SPX4 | -- | -- | BnaC9.SPX4 | -- | -- |
| tPCK4 | U | AtSPX-MFS1 At4g22990 | Bra013668 | -- | -- | Bol014972 | -- | -- | BnaA1.SPX-MFS1 | -- | -- | BnaC1.SPX-MFS1 | -- | -- |
| tPCK5 | P | AtSPX-MFS2 At4g11810 | -- | -- | -- | Bol008313 | -- | -- | -- | -- | -- | -- | -- | -- |
| tPCK7 | D | AtSPX-MFS3 At1g63010 | -- | Bra036635 | Bra027004 | -- | -- | Bol029704 | -- | -- | BnaA9.SPX-MFS3a | -- | -- | -- |
| tPCK1 | A | AtNLA1 At1g02860 | Bra033354 | -- | -- | Bol040781 | -- | Bol018410 | -- | -- | -- | -- | -- | -- |
| tPCK3 | J | AtNLA2 At2g38920 | -- | Bra017094 | Bra000106 | -- | Bol025322 | Bol020396 | -- | -- | BnaA3.NLA2 | -- | -- | -- |
| tPCK2 | F | AtPHO1 At3g23430 | Bra014948 | Bra023727 | -- | Bol037279 | Bol028738 | -- | BnaA7.PHO1 | BnaA1.PHO1 | -- | BnaC7.PHO1 | -- | -- |
| tPCK6 | E | AtPHO1;H1 At1g68740 | Bra004334 | Bra038357 | Bra004017 | Bol023994 | Bol028648 | Bol003325 | BnaA7.PHO1;H1b |  | BnaA7.PHO1;H1a | BnaC6.PHO1;H1b | BnaC2.PHO1;H1 | -- |
| tPCK7 | K | AtPHO1;H2 At2g03260 | Bra024782 | Bra026565 | Bra017393 | Bol007225 | Bol015191 | Bol032526 | -- | -- | -- | -- | -- | -- |
| tPCK1 | A | AtPHO1;H3 At1g14040 | Bra019690 | -- | Bra026863 | Bol001609 | -- | Bol031467 | BnaA6.PHO1;H3b | -- | BnaA9.PHO1;H3a | BnaC5.PHO1;H3 | -- | BnaC8.PHO1;H3a |
| tPCK4 | U | AtPHO1;H4 At4g25350 | -- | -- | Bra010468 | -- | Bol042228 | Bol002787 | -- | -- | -- | -- | -- | BnaC8.PHO1;H4 |
| tPCK7 | K | AtPHO1;H5 At2g03240 | Bra024782 | Bra026565 | Bra017392 | Bol007225 | Bol015191 | Bol032526 | -- | -- | -- | -- | -- | -- |
| tPCK7 | K | AtPHO1;H6 At2g03250 | Bra024782 | Bra024782 | Bra017393 | Bra017393 | Bol007225 | Bol032526 | -- | -- | -- | -- | -- | -- |
| tPCK1 | B | AtPHO1;H7 At1g26730 | Bra024691 | Bra016284 | -- | Bol023711 | Bol008726 | -- | -- | -- | -- | -- | -- | -- |
| tPCK1 | B | AtPHO1;H8 At1g35350 | -- | -- | -- | -- | -- | -- | -- | -- | -- | -- | -- | -- |
| tPCK7 | L | AtPHO1;H9 At3g29060 | -- | Bra033081 | Bra036222 | -- | Bol033191 | Bol032272 | -- | BnaA2.PHO1;H9 | BnaA9.PHO1;H9 | -- | -- | BnaC9.PHO1;H9 |
| tPCK6 | E | AtPHO1;H10 At1g69480 | Bra004383 | -- | -- | Bol023935 | -- | -- | BnaA7.PHO1;H10 | -- | -- | BnaC6.PHO1;H10 | -- | -- |

The data were downloaded from the *Brassica Database*. a: tPCK Chr: Chromosome of translocation, Proto-Calepineae Karyotype, ancestral genome of *Brassica* species; b LF: Less fractioned subgenome; c MF(MF1 and MF2) more fractioned subgenome.
