# Supplementary material for: Genome-Wide Identification and Characterization of SPX Domain-Containing Members and Their Responses to Phosphate Deficiency in Brassica napus
Source: Front Plant Sci. 2017 Jan 25;8:35. doi: 10.3389/fpls.2017.00035 (PMC5263162; doi:10.3389/fpls.2017.00035)
Supplement: Supplementary file 7 [file Table_3.DOCX]

**Supplementary Table 3.** Normalized FPKM of detected BnaSPXs in RNA-seq analysis

|  |  | **FPKM** | | | |
| --- | --- | --- | --- | --- | --- |
| **Gene locus** | **Gene name** | **HP-S** | **LP-S** | **HP-R** | **LP-R** |
| BnaA02g04730D | *BnaA2.SPX1* | 0.340506 | 298.599 | 0.670771 | 293.359 |
| BnaA03g07930D | *BnaA3.SPX1* | 7.87629 | 785.129 | 19.8484 | 676.818 |
| BnaC03g10000D | *BnaC3.SPX1* | 0.021268 | 1.97427 | 0.07631 | 1.62792 |
| BnaA03g22680D | *BnaA3.SPX2* | 1.6157 | 29.8695 | 2.33174 | 25.8272 |
| BnaC03g26700D | *BnaC3.SPX2* | 7.55608 | 142.965 | 13.1011 | 135.564 |
| BnaA03g21050D | *BnaA3.SPX3* | 0.007884 | 1.53653 | 0 | 6.85037 |
| BnaA04g26060D | *BnaA4.SPX3* | 0.103948 | 99.3063 | 0.420862 | 46.878 |
| BnaC03g25110D | *BnaC3.SPX3* | 0.008031 | 7.69955 | 0 | 15.3119 |
| BnaC04g50120D | *BnaC4.SPX3* | 0.20748 | 93.1428 | 0.189586 | 42.25 |
| BnaA10g18780D | *BnaA10.SPX4* | 1.33266 | 1.49367 | 10.2841 | 9.48152 |
| BnaC09g42510D | *BnaC9.SPX4* | 3.38697 | 6.05487 | 15.4408 | 10.5228 |
| BnaA01g12800D | *BnaA1.SPX-MFS1* | 1.09945 | 2.30249 | 0.050091 | 0.057671 |
| BnaC01g14580D | *BnaC1.SPX-MFS1* | 1.59912 | 2.97796 | 0.372444 | 0.53529 |
| BnaA09g21340D | *BnaA9.SPX-MFS2* | 0.007094 | 0 | 0 | 0.046934 |
| BnaC09g23750D | *BnaC9.SPX-MFS2* | 0 | 0.024911 | 0 | 0.097318 |
| BnaA09g12960D | *BnaA9.SPX-MFS3a* | 0.87262 | 1.44373 | 7.03289 | 7.23238 |
| BnaC09g12880D | *BnaC9.SPX-MFS3* | 1.83219 | 3.15784 | 7.07979 | 7.54364 |
| BnaA09g51130D | *BnaA9.NLA1* | 0.729404 | 1.13205 | 7.83137 | 3.41949 |
| BnaA10g01450D | *BnaA10.NLA1* | 0.080555 | 0.260919 | 6.24702 | 3.45585 |
| BnaC05g01480D | *BnaC5.NLA1* | 0.157332 | 0.310384 | 19.1405 | 6.58416 |
| BnaC08g45940D | *BnaC8.NLA1* | 0.353216 | 0.237987 | 12.8399 | 4.70203 |
| BnaC04g46040D | *BnaC4.NLA2* | 2.64162 | 3.49919 | 0.364915 | 0.483649 |
| BnaA01g23840D | *BnaA1.PHO1* | 0.32582 | 1.19723 | 66.6057 | 86.6088 |
| BnaA07g06530D | *BnaA7.PHO1* | 0.324725 | 0.752782 | 61.4333 | 35.231 |
| BnaC01g30800D | *BnaC1.PHO1* | 0.208527 | 1.23425 | 45.0555 | 59.4197 |
| BnaC07g08080D | *BnaC7.PHO1* | 0.143905 | 0.257765 | 22.5396 | 13.5398 |
| BnaA07g24450D | *BnaA7.PHO1;H1a* | 0 | 0.560342 | 0.056386 | 1.83658 |
| BnaA07g27390D | *BnaA7.PHO1;H1b* | 0.18826 | 13.0807 | 10.6931 | 22.7299 |
| BnaC02g18430D | *BnaC2.PHO1;H1* | 0.057504 | 7.23186 | 0.946902 | 1.03792 |
| BnaC06g25630D | *BnaC6.PHO1;H1a* | 0 | 0.013195 | 0.112344 | 0.453835 |
| BnaC06g30370D | *BnaC6.PHO1;H1b* | 0.611221 | 21.5077 | 14.6226 | 36.3661 |
| BnaA06g08940D | *BnaA6.PHO1;H3b* | 6.83648 | 7.78182 | 14.974 | 4.72574 |
| BnaA09g45870D | *BnaA9.PHO1;H3a* | 0.006307 | 0.0124974 | 1.52513 | 1.30446 |
| BnaA09g45890D | *BnaA9.PHO1;H3b* | 0.080054 | 0.532153 | 0.003977 | 0 |
| BnaC05g10300D | *BnaC5.PHO1;H3* | 0.903206 | 0.386092 | 7.92508 | 4.90997 |
| BnaC08g39940D | *BnaC8.PHO1;H3a* | 0.096975 | 0.125021 | 5.01377 | 5.11111 |
| BnaC08g39950D | *BnaC8.PHO1;H3b* | 4.8904 | 18.4553 | 0.027056 | 0.086645 |
| BnaC07g39540D | *BnaC7.PHO1;H4* | 0.004303 | 0.00892729 | 0.217239 | 0.267597 |
| BnaA02g26710D | *BnaA2.PHO1;H5* | 1.90601 | 3.1475 | 4.18118 | 1.83237 |
| BnaA06g33790D | *BnaA6.PHO1;H5* | 0.02547 | 0.0778153 | 1.1034 | 0.227173 |
| BnaA09g19200D | *BnaA9.PHO1;H5a* | 1.27745 | 0.586716 | 4.04777 | 3.85787 |
| BnaA09g19210D | *BnaA9.PHO1;H5b* | 0.057501 | 0.0363131 | 0.657092 | 0.638638 |
| BnaC02g34860D | *BnaC2.PHO1;H5* | 1.06273 | 1.97733 | 9.27967 | 5.07059 |
| BnaC07g22160D | *BnaC7.PHO1;H5* | 0.282029 | 0.366236 | 2.09366 | 1.03414 |
| BnaC01g13200D | *BnaC1.PHO1;H8* | 5.74048 | 10.3149 | 0.083501 | 0.058407 |
| BnaA09g29180D | *BnaA9.PHO1;H8* | 0.002505 | 0 | 0 | 0.006095 |
| BnaA08g19850D | *BnaA8.PHO1;H8* | 19.6081 | 18.5978 | 0.458747 | 0.256455 |
| BnaA02g29650D | *BnaA2.PHO1;H9* | 0.002103 | 0 | 0 | 0 |
| BnaC06g30650D | *BnaC6.PHO1;H10* | 0.276255 | 0.357607 | 0.45567 | 0.380107 |
| BnaA07g27980D | *BnaA7.PHO1;H10* | 0 | 0.0172326 | 0.003814 | 0.043664 |
