# Supplementary material for: Genome-Wide Identification and Characterization of SPX Domain-Containing Members and Their Responses to Phosphate Deficiency in Brassica napus
Source: Front Plant Sci. 2017 Jan 25;8:35. doi: 10.3389/fpls.2017.00035 (PMC5263162; doi:10.3389/fpls.2017.00035)
Supplement: Supplementary file 8 [file Table_4.DOCX]

**Supplementary Table 4** Primers used in the present study

| Primer name | Forward sequence (5' -3') | Reverse sequence (5' -3') |
| --- | --- | --- |
| Primers used in vector construction | |  |
| OeBnaA2. SPX1 | GGCGCGCCCGAACGAATAACCAAATTG | TCTAGACTATTTGGCTTCTTGCTCTAACAA |
| OeBnaC3. SPX1 | GGCGCGCCGAAGGATGGTTTTAGTGAGGTC | TCTAGACTATTTTGCTTCTTGCTCCAAC |
| BnaSPX1s-GFP | TTAATTAAGCCACCATGAAGTTCGGTAAGAG | GGCGCGCCGTTTGGCTTCTTGCTCCAAC |
| BnaSPX2s-GFP | TTAATTAAGCCACCATGAAATTCGGCAAGAG | GGCGCGCCGTTCTGCTACTTGTTCCAG |
| Primers used in qRT-PCR analysis | |  |
| qBnaA2.SPX1 | CCAAGCAGAGCTCTCAGAG | GAATCTTCTTCCATGTCTCA |
| qBnaC3.SPX2 | GACCTGCTCAACACATTTGTG | GGTTTCTGAGTTGACAGTACCAG |
| qBnaC3.SPX3 | GTGAGTTCAGCGGCTGCG | CTAACGACATCTTCCTAGAACGAC |
| qBnaC9.SPX4 | CTCCATCAACCCTTCTTC | GAAGAAGTCTGAGCAGAGATC |
| qBnaC4.SPX3 | AATGCCTTCTTCGTGGAG | CTCTTGTCGTACTTCTTCAG |
| qBnaA3.SPX1 | GCTCTGAACTACACAGGATT | CTCCATGAACTTGTGCTCC |
| qBnaC3.SPX1 | GCTCATCAAGGAGTCCGAG | CAGAGCAGCGATCGTACTC |
| qBnaA10.SPX4 | GACTCTCACGACGGTGTAAGGC | CTTTCCTTCAACTCCTGCTGTAG |
| qBnaC3.SPX3 | GTCTGCGGCGGTGAGTTC | GGAAGGGATGTGTATCGGTTG |
| qBnaA3.SPX3 | GGTGCTTACTCGCTTCCG | GATAAGTCCATTTCCCGTGTG |
| qBnaA3.SPX2 | ATCCAAAAGGTTCTGCAAGAG | GTTGGCTCTGATTTGTCTTCG |
| BnACTIN2 | ACAGTGTCTGGATCGGTGGTTC | TGCCTCATCATACTCAGCCTTG |
| AtACTIN7 | GGAGCTGAGAGATTCCGTTG | GGTGCAACCACCTTGATCTT |
| qAtPHT1;1 | CCTTTGGGTTCCTATATGCG | TAACCTCAGCCTCACCAGAG |
| qAtPHT1;4 | TCAATGGCGTTGCCTTCTGT | ATCACCAAGCCACCCGAAA |
| qAtPHO1 | TGGTTCTCCGGAACAAGAAC | TGACTTCAAGTGACGCCAAG |
| qAtPAP10 | TCCTGTTGATGATTCTCCTTCTTG | ATTCATTTATTTGGATGGTTGTTCA |
| qAtPHO2 | CGAACACGATATATGTGCG | CATTGGTGGCTCATGAGG |
| qOE-BnaA2.SPX1 | CTCAAGCTCATCACCTCCACAACTC | GGACAATCCTAGGGAACACTCGG |
| qOE-BnaC3.SPX1 | TCTCAAGCTCATCGACTCCAAAAG | GATGATGTACTCTTCCTCCTTCTCG |
| qAtNLA | GGCTCAGAGGCAACAGTCG | CGCTTGTCCTTGCCTAGACTC |
| qAth-miR399e | CTCTATTGGCAGTGGAAGTTGATGACC | ACGTTAGTGAAGCATTGCGAGGC |
| qAth-miR399f | GCATTACAGGGCAAGATCACCATTGG | GCGCAAGAGAATTACCGGGCAAATC |
| qAth-miR827 | GCAACCCTTGAATGTGTTTG | AACCACGAAAGAGTTTGTTGATGG |
| qAtSQD1 | GGGACTCTCAACGTTCTCTTTG | CCCATCGTCCCAAGTTTTAC |
| qAtPHR1 | GCTTGTCAAGGAATCTGAGGC | GAACTTCCACTCCTGATCTCC |
